# Regulation of Metabolism by Mitochondrial MUL1 E3 Ubiquitin Ligase

**DOI:** 10.3389/fcell.2022.904728

**Published:** 2022-06-29

**Authors:** Lucia Cilenti, Rohit Mahar, Jacopo Di Gregorio, Camilla T. Ambivero, Matthew E. Merritt, Antonis S. Zervos

**Affiliations:** ^1^ Burnett School of Biomedical Sciences, University of Central Florida College of Medicine, Orlando, FL, United States; ^2^ Department of Biochemistry and Molecular Biology, University of Florida, Gainesville, FL, United States

**Keywords:** MUL1, Akt2, HIF-1α, mitochondrial metabolism, metabolic flux

## Abstract

MUL1 is a multifunctional E3 ubiquitin ligase that is involved in various pathophysiological processes including apoptosis, mitophagy, mitochondrial dynamics, and innate immune response. We uncovered a new function for MUL1 in the regulation of mitochondrial metabolism. We characterized the metabolic phenotype of MUL1(−/−) cells using metabolomic, lipidomic, gene expression profiling, metabolic flux, and mitochondrial respiration analyses. In addition, the mechanism by which MUL1 regulates metabolism was investigated, and the transcription factor HIF-1α, as well as the serine/threonine kinase Akt2, were identified as the mediators of the MUL1 function. MUL1 ligase, through K48-specific polyubiquitination, regulates both Akt2 and HIF-1α protein level, and the absence of MUL1 leads to the accumulation and activation of both substrates. We used specific chemical inhibitors and activators of HIF-1α and Akt2 proteins, as well as Akt2(−/−) cells, to investigate the individual contribution of HIF-1α and Akt2 proteins to the MUL1-specific phenotype. This study describes a new function of MUL1 in the regulation of mitochondrial metabolism and reveals how its downregulation/inactivation can affect mitochondrial respiration and cause a shift to a new metabolic and lipidomic state.

## Introduction

The primary function of mitochondria is to meet the energy demand by providing ATP through oxidative phosphorylation (OXPHOS); in addition, they have a central role in cellular homeostasis, cell death, and the regulation of metabolism (Herst et al., 2017; Annesley and Fisher, 2019; Rossmann et al., 2021). Mitochondria can modify their bioenergetic and biosynthetic functions to meet the metabolic demands of the cell, in response to changes in the physiological environment (nutrient stress) or low O_2_ levels (hypoxia) (Fuhrmann and Brune, 2017; McElroy and Chandel, 2017; Glancy et al., 2020). The regulation of metabolism requires continuous and effective communication between the mitochondria with the rest of the cell, which involves numerous proteins with diverse functions (Weinberg and Chandel, 2015). MUL1 (also known as Mulan, MAPL, GIDE, and HADES) is a mitochondrial E3 ubiquitin ligase and one of only three E3 ligases found in mitochondria (the other two are MARCH5 and RNF185) (Li et al., 2008; Zhang et al., 2008; Braschi et al., 2009; Jung et al., 2011; Tang et al., 2011; Ambivero et al., 2014; Cilenti et al., 2014; Kim et al., 2016). MUL1 is anchored in the outer mitochondrial membrane (OMM) with two transmembrane domains, a large intermembrane domain (IMD), and a RING finger domain facing the cytoplasm, which is responsible for its ligase function. Based on its topography, MUL1 can convey changing conditions within the mitochondria to ubiquitinate specific substrates in the cytoplasm. MUL1 is able to K48- or K63-ubiquitinate, as well as SUMOylate, a number of specific substrates and its function has been implicated in the regulation of mitophagy, cell death, mitochondrial dynamics, and innate immune response (Zhang et al., 2008; Braschi et al., 2009; Jenkins et al., 2013; Cilenti et al., 2014; Yun et al., 2014; Li et al., 2015; Prudent et al., 2015; Ni et al., 2017; Cilenti et al., 2020; Puri et al., 2020). We have previously shown that MUL1 through UBXN7 can regulate the HIF-1α protein level to support a glycolytic phenotype in cells (Cilenti et al., 2020; Di Gregorio et al., 2021). In addition, the AKT protein, which is implicated in the regulation of metabolism, is also a known substrate of MUL1 (Bae et al., 2012; Kim et al., 2015; Kim et al., 2017). Here, we show that MUL1, through K48 polyubiquitination, co-regulates both Akt2 and HIF-1α protein level to maintain a normal metabolic state. Inactivation of MUL1 leads to the accumulation of both Akt2 and HIF-1α proteins and has a profound effect on glycolysis, lipid metabolism, mitochondrial anaplerotic fluxes, and pyruvate cycling and defines a unique metabolic state. In summary, we identified and characterized a new function of mitochondrial MUL1 E3 ubiquitin ligase in mitochondrial respiration and the regulation of metabolism. Furthermore, we delineated that the MUL1 involvement in metabolism is mediated by the concurrent regulation of Akt2 kinase and HIF-1α transcription factor.

## Materials and Methods

### Cell Culture and Chemicals

HEK293 wild type (WT), HEK293 MUL1(−/−), and HEK293 Akt2(−/−) cells were grown using Dulbecco’s modified Eagle’s medium (DMEM high glucose, sodium pyruvate) supplemented with 10% fetal calf serum (Atlanta Biologicals), 2 mM l-glutamine, 50 units/ml penicillin, and 50 μg/ml streptomycin (Thermo Fisher Scientific, United States). HeLa cells were maintained in Dulbecco’s modified Eagle’s medium supplemented with 10% fetal bovine serum (Atlanta Biological), 50 units/ml penicillin, and 50 μg/ml streptomycin. Chemicals: perifosine, chetomin, DMOG, oligomycin, FCCP, antimycin A, and rotenone (SIGMA) were dissolved in DMSO, stored at -80°C, and used at the indicated concentrations. XF DMEM media pH 7.4, glucose, pyruvate, and glutamine solutions were obtained from Agilent Technologies. DMSO (0.1%) was used as a vehicle control.

### SDS-PAGE and Western Blot Analysis

Control (untreated) and cells treated with perifosine**,** chetomin, or DMOG were lysed using a Triton X-100-based lysis buffer (1% Triton X-100, 10% glycerol, 150 mM NaCl, 20 mM tris (pH 7.5), 2 mM EDTA) in the presence of protease inhibitors (Thermo Fisher Scientific). Approximately 40 μg of whole cell extract was resuspended in the SDS sample buffer, boiled for 4 min, and analyzed by SDS-PAGE (12% or 15% gels) and then transferred onto PVDF membranes (Genesee) using a semi-dry cell transfer blot (Bio-Rad). Nonfat dry milk (4%) or 5% BSA (for Akt2 and phospho-specific antibodies) in TBST buffer (25 mM tris–HCl, pH 8.0, 125 mM NaCl, 0.1% Tween 20) was used to block the nonspecific binding of the membrane. The membranes were incubated with the indicated primary antibodies: HIF-1α (Bioss Antibodies, 1:2,000), AKT (pan), Akt1, Akt2, Akt3, phospho-Akt-T308, phospho-GSK-3β-S9, ULK1 (Cell Signaling Technology 1:2,000), GLUT1 (ABclonal 1:2,000), MFN2, and β-actin (Santa Cruz Biotechnology, 1:3,000). MUL1 and UBXN7 rabbit polyclonal antibodies were homegrown and used at a 1:5,000 dilution. Secondary peroxidase-conjugated goat anti-rabbit or goat anti-mouse antibodies (Jackson ImmunoResearch) were used at a 1:10,000 dilution. The membrane was visualized by enhanced chemiluminescence (ECL) (Thermo Fisher Scientific).

### Generation of HEK293 *Akt2(−/−)* and HeLa *Mul1*(−/−) Using CRISPR/Cas9 Gene Editing

To ablate *Akt2* expression, the target sequence 5′–GAC​CCC​ATG​GAC​TAC​AAG​TG–3′ located in exon 4 was selected using the CRISPOR program (doi: 10.1186/s13059-016-1,012-2; http://crispor.tefor.net) and cloned into the pSpCas9(BB)-2A-GFP (PX458) vector (Addgene), as previously described (Ran et al., 2013). To knockout *Mul1* in HeLa cells, a similar method was used with the specific target sequence 5′–GCC​GCC​GTC​ATG​GAG​AGC​GG–3′ in exon 1. The resulting vectors (PX458-*Akt2-target* and PX458-*Mul1-target*) or the empty PX458 control vector was transfected into HEK293 or HeLa cells, respectively. Forty-eight hours later, single GFP-positive cells were sorted into 96-well plates using a FACS ARIA II sorter (BD Biosciences). The clones were expanded, and Akt2 or MUL1 protein expression was monitored by the western blot analysis. In addition, genomic DNA was isolated and used for PCR amplification and DNA sequencing to verify the deletion of the target sequence surrounding exon 4 of the *Akt2* gene or exon 1 of the *Mul1* gene. Three independent HEK293 Akt2(−/−) and three HeLa MUL1(−/−) clones, as well as two HEK293 or HeLa WT control clones (transfected with a PX458 empty vector), were chosen for further experiments. The HEK293 MUL1(−/−) cells have been previously described (Steffen et al., 2017; Cilenti et al., 2020; Di Gregorio et al., 2021).

### Glycolytic Stress Assay

To monitor glycolysis in the cell lines, the glycolysis stress test was performed using an XFe24 extracellular flux analyzer (Agilent Technologies). HEK293 WT, MUL1(−/−), and Akt2(−/−) cells were seeded in triplicate on poly-lysine D-coated XFe24 microplates at a density of 60,000 cells per well in the assay medium (XF DMEM pH 7.4 containing 2 mM glutamine and 1 mM sodium pyruvate without glucose) for 1 h in a CO_2_-free incubator. Extracellular acidification rate (ECAR) was measured under basal conditions and again after sequential injections of glucose (10 mM) in port A, ATP synthase inhibitor oligomycin (1.5 μM) in port B, and the glycolysis inhibitor 2-deoxyglucose (2-DG) (300 mM) in port C. Basal glycolysis was assessed by recording three measurements, following the addition of each compound by a 3-2-3 mix/measurement cycle. Glycolysis is the ECAR after the addition of glucose. Glycolytic capacity is the increase in ECAR after the injection of oligomycin following glucose. Glycolytic reserve is the difference between the glycolytic capacity and glycolysis. Data analysis was performed using Report Generator software for glycolysis stress test (Agilent Technologies).

### Mitochondrial Stress Assay

Mitochondrial stress assay was performed using an XFe24 extracellular flux analyzer (Agilent Technologies) following the workflow provided by the manufacturer’s instructions. Briefly, for oxygen consumption rate (OCR) measurements, HEK293 WT and MUL1(−/−) cells were seeded in triplicate on poly-lysine D-coated XF24 microplates at a density of approximately 60,000 cells per well in the assay medium (XF DMEM medium pH 7.4 supplemented with 10 mM glucose, 2 mM glutamine, and 1 mM pyruvate), followed by incubation at 37°C in a CO_2_-free incubator for 60 min. Three baseline measurements were recorded before the injection of the following compounds: 1.5 μM of oligomycin in port A, 1.0 μM of FCCP in port B, and 0.5 μM rotenone/antimycin in port C. Data analysis was performed using Cell Mito Stress Test Report Generator software (Agilent Technologies).

### Stable Isotope Tracer Experiment With Cells for NMR Spectroscopy

For NMR analysis, approximately 20 × 10^6^ HEK293 WT, MUL1(−/−), or Akt2(−/−) cells were grown to confluency following treatment with perifosine or chetomin inhibitors, as well as with the HIF-1α activator DMOG, and DMSO was used as a vehicle control. A freshly prepared [U-^13^C]glucose tracer containing medium (glucose free DMEM with 10% dialyzed FBS and 15 mM [U-^13^C]glucose) was added to the cells for 6 h at 37°C. Cells were scraped from the plates, washed twice with cold PBS, and immediately snap-frozen in liquid nitrogen (Yuan et al., 2019).

### Extraction of the Metabolites and NMR Analysis

Cell pellets were bead-homogenized in 1 ml acetonitrile:isopropanol:water (3:3:2, v:v:v) and centrifuged at 10,000 × g for 15 min at 4°C, and the supernatant was removed and dried in a speedvac. The dried cell extracts of each sample were resuspended in 0.5 ml of acetonitrile:water (1:1, v:v) solution, vortexed, and centrifuged for 15 min at 10,000 × g at 4°C. The supernatant was transferred to a new tube and dried in a speedvac. The dried extract of each cell pellet was re-suspended in phosphate buffer prepared in deuterium oxide (D_2_O) for NMR analysis. The final volume of the sample (50 μl) consisted of 90% (v/v) deuterated 50 mM sodium phosphate buffer (pH 7), with 2 mM ethylene diamine tetra-acetic acid (EDTA), whereas 10% (v/v) was occupied by 5 mM D_6_-4,4-dimethyl-4-silapentane-1-sulfonic acid (DSS) and 0.2% sodium azide (NaN_3_) in D_2_O (Miccheli et al., 2006; Chen et al., 2021).

### NMR Spectroscopy and Data Processing

^1^H-NMR spectra were collected on an 800 MHz NMR, equipped with a 5 mm TXI CryoProbe and an Avance III console (Bruker Biospin), using TopSpin software (version 3.6.3). The ^1^H-NMR spectra were acquired using a noesypr1d pulse sequence consisting of a 1 s relaxation delay (d1) and a mixing time of 100 ms. A 4 s acquisition time (AQ) over a spectral width (sw) of 12 ppm gave a final time-to-repeat of 5.1 s. A total of 64 scans were acquired for each spectrum. The conventional ^1^H-decoupled ^13^C-NMR spectra were acquired for each sample at 150.13 MHz using a^13^C-optimized 1.5 mm high-temperature superconducting (HTS) probe on an Agilent NMR system with a magnetic field strength of 14 T. ^13^C-NMR spectra were recorded using an acquisition time (AQ) of 1.5 s, a relaxation delay (d1) of 1.5 s, a flip angle of 45°, and an acquired size (TD) of 54 k with a spectral width of 250 ppm (Ramaswamy et al., 2013). All NMR spectra were acquired at room temperature (25°C). NMR data processing was performed in MestReNova software (*v*14.0.1-23284, Mestrelab Research S.L.). ^1^H-NMR spectra were Fourier-transformed (FT) with a line-broadening factor of 0.5 Hz, zero filling to 65,536 data points, and baseline correction with the spline method. ^13^C-NMR spectra were processed with the following processing parameters: zero filling to 128k data points, an exponential line broadening of 0.5 Hz, manual phase correction, and Whittaker smoother method for baseline correction.

### ^13^C-NMR Analysis of the Glutamate Isotopomers

The positional ^13^C-isotopomer distribution pattern of glutamate was determined by ^13^C-NMR. ^13^C-NMR resonances of various isotopomers of glutamate are identified from the ^13^C–^13^C J-coupling constants and quantified by line fitting to the glutamate resonances at C2, C3, C4, and C5 positions. The ^13^C-labeling was described first by the position of the ^13^C label and then by the multiplicity of the resonance and its origin. For example, C2S would denote the isotopomer of glutamate labeled only at the C2 position. The descriptor C2D12 describes the resonance of the C2 position that is split into a doublet (D) from coupling to an adjacent ^13^C label at the C1 position. The descriptor C2D23 denotes the resonance of the C2 position that is split into a doublet (D) from coupling to an adjacent ^13^C label at the C3 position. When a^13^C-labeled C2 is flanked by two other neighboring ^13^C-labeled positions (C1 and C3), the resonance is split twice by the J-coupling into a doublet of doublets or a quartet (Q), denoted C2Q. Similarly, other carbon resonances of glutamate were assigned, and peak areas were extracted for each of the isotopomers.

### Fluxomic Analysis Using ^13^C-Isotopomer Data in tcaCALC

Metabolic flux relative to Krebs cycle turnover was calculated from the ^13^C-isotopomers of glutamate observed at carbon positions 2, 3, 4, and 5 in the ^13^C-NMR spectra of each cell sample. The tcaCALC program in MATLAB was utilized to perform an isotopomer analysis to estimate relative pathway fluxes (Alger et al., 2021). The metabolic model provides the best fit to our ^13^C-NMR data by calculating the relative fluxes of pyruvate dehydrogenase (PDH), pyruvate carboxylase (Y_PC_), pyruvate kinase (PK), and anaplerosis leading to succinyl-CoA (Ys). All flux rates are referenced to a citrate synthase (CS) flux of 1 and is equivalent to Kreb’s cycle flux. The input file contains the peak area ratios with initial parameters for PDH, PK, Y_PC_, and Ys fluxes of 0.2, 03, 0.1, and 0.2, respectively.

### LC-MS Analysis for Metabolomics and Lipidomic

Cell pellets of HEK293 WT or MUL1(−/−) cells were subjected to the Folch extraction procedure, which resulted in two phases, i.e., aqueous phase and chloroform phase, containing polar metabolites and non-polar lipids, respectively. The aqueous phase was analyzed using the Thermo Q-Exactive Orbitrap mass spectrometer with a Dionex UHPLC and an autosampler. All samples were analyzed in positive and negative heated electrospray ionization (HESI) with a mass resolution of 35,000 at m/z 200 as separate injections. Separation was achieved on an ACE 18-pfp 100 × 2.1 mm, 2 µm column with mobile phase A as 0.1% formic acid in water and mobile phase B was acetonitrile. The flow rate was 350 μl/min with a column temperature of 25°C. A 4 µl sample solution was injected for negative and 2 µl for positive ion mode LC-MS analysis. MZmine software was used to identify features and deisotopes, align features, and perform gap filling. The metabolomics data were searched against the SECIM’s internal retention time metabolite library. LC-MS analysis for lipidomics was performed on the chloroform phase of the Folch extracted cell samples, utilizing a Thermo Q-Exactive Orbitrap mass spectrometer with a Dionex UHPLC and an autosampler. All samples were analyzed in the HESI source with a mass resolution of 35,000 at m/z 200. LC separation was achieved on an Acquity BEH C18 1.7 µm, 100 × 2.1 mm column. The mobile phase A (60:40 acetonitrile:10 mM ammonium formate with 0.1% formic acid water) and mobile phase B (90:8:2 2-propanol: acetonitrile: 10 mM ammonium formate with 0.1% formic acid in water) were used for elution of the lipids. The flow rate was 500 μl/min, and the column temperature was maintained at 50°C. Lipidomics data were analyzed using LipidMatch software (Koelmel et al., 2017).

### Metabolomics Data Analysis

The web-based tool MetaboAnalyst (https://www.metaboanalyst.ca) was used for the metabolomic data analysis, interpretation, and integration of the joint metabolic pathway analysis of genomics and metabolomic data. For multivariate statistical analysis, LC-MS data from positive and negative ion modes were imported into MetaboAnalyst software and subjected to normalization by the sum of the intensities, data transformation *via* log transformation, and pareto scaling. Joint metabolic pathway analysis was performed with gene and metabolite using uniport protein ID and HMDB ID, respectively. The fold change for MUL1(−/−) compared to that for WT genes and metabolites was used as an input to perform the joint pathway analysis. Metabolic pathways containing both metabolites and genes were used for the integrative pathway analysis along with Fisher’s exact test for the enrichment analysis. Simultaneously, an interactive network of pathway analysis, showing the connections between pathways and an individual pathway, was carried out, using significantly different metabolites between HEK293 WT and MUL1(−/−) cells using results from both positive and negative mode ionization. A quantitative metabolomic panel of significantly different metabolites between HEK293 WT and MUL1(−/−) cells was created, employing statistical analysis *via t-*test in MetaboAnalyst (Chong et al., 2019). Box-and-whisker plots were prepared in GraphPad prism, which displayed the differential level of significantly different metabolites between HEK293 WT and MUL1(−/−) cells.

### Lipidomic Data Analysis

High-resolution LC-MS/MS-based lipidomic profiling of lipid classes was carried out using the normalized individual lipid intensity to the total lipid intensity of each sample. The values for each lipid class and the top 10 most abundant lipids in each class are expressed as mean ± SEM (n = 4). Lion term enrichment analysis of HEK293 WT versus MUL1(−/−) cells in the ranking mode was carried out by lipid ontology (LION): a web-based interface used for the identification of lipid-associated terms in lipidomes (Molenaar et al., 2019). The cut-off value of significantly up and down lipids in HEK293 MUL1(−/−) with respect to WT cells (*p* < 0.05) was determined, and the data were scaled with the enrichment (−log FDR *q*-values).

### RNA Sequencing Analysis

Standard RNA sequencing for the gene profiling expression of protein-coding sequences (mRNA) was performed using HEK293 WT and MUL1(−/−) cells. Approximately 10 million HEK293 WT and MUL1(−/−) cells (*n* = 3 per group) were used for library preparation, DNA sequencing, and data analysis (GENEWIZ Global). Using DESeq2, a comparison of gene expression between the defined groups of samples was performed. The Wald test was used to generate *p* values and log2 fold changes. Genes with an adjusted *p* value <0.05 (*p* < 0.05) and absolute log2 fold change >1 were labeled as differentially expressed genes. Significantly differentially expressed genes were clustered by their gene ontology, and the enrichment of gene ontology terms was tested using the Fisher exact test. Heatmaps of the top 50, as well as genes specifically involved in lipid, glucose, carbohydrate, glutathione, and retinoic acid metabolomic processes, were analyzed using RStudio and Heatmap.2 software (Chen et al., 2016).

### Statistical Analysis

All quantitative data were expressed as mean ± SD or ±SEM of three or four independent experiments. Following the western blot analysis, the optical densities of blot bands were determined using ImageJ software. Protein/β-actin ratios were obtained from the densitometry data, and the differences among groups were analyzed by one-tailed Student’s *t* test. A value of *p* ≤ 0.05 was considered significant. All Seahorse data were analyzed using Report Generator software that automatically calculates and reports the assay parameters of the Agilent Seahorse XFe24 specific for each assay (glycolysis stress test or cell mito stress) (Agilent Technologies). For data analysis (NMR spectroscopy, LC-MS metabolomic, lipidomic, and RNA-seq), a value of *p* ≤ 0.05 was considered significant.

## Results

### MUL1 E3 Ubiquitin Ligase Regulates the Protein Level of Several Substrates With Diverse Function

**FIGURE 1:**
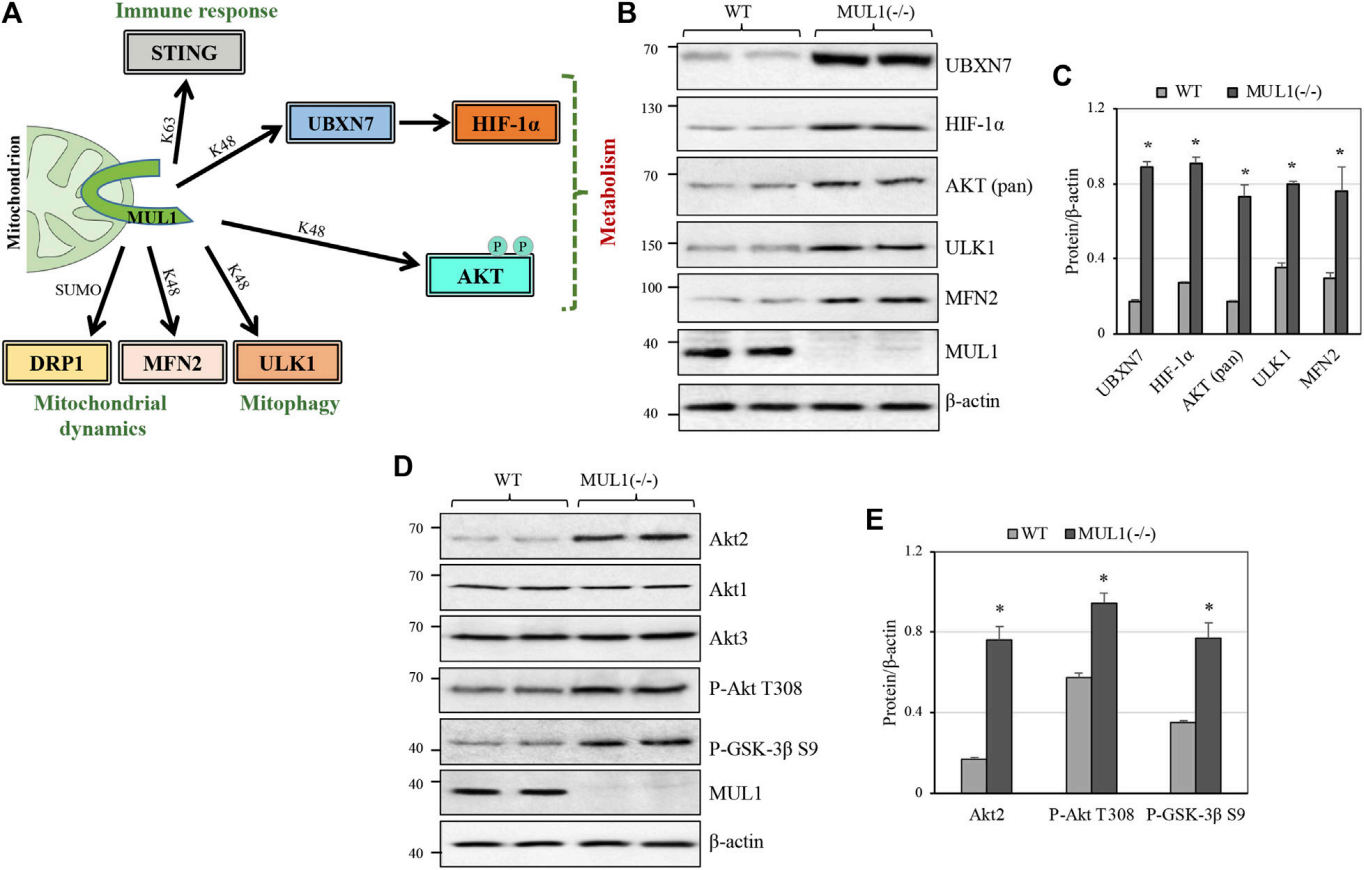
MUL1 is a multifunctional E3 ubiquitin ligase, and its inactivation leads to the accumulation of various substrates. **(A)** MUL1 is a mitochondrial E3 ligase with a diverse role in immune response, mitochondrial dynamics/mitophagy, and metabolism (present study). MUL1 mediates its function through K63, or K48-ubiquitination, as well as SUMOylation of the specific substrates shown. **(B)** Western blot analysis to monitor the protein level of several known MUL1 K48 ubiquitination substrates in two independent clones of HEK293 WT and MUL1-(−/−) cells. β-actin was used to verify equal loading in each lane. **(C)** Graph represents the densitometrical analysis of the proteins shown in panel (B), normalized against β-actin. Results shown as means ± SD of three independent experiments. *, *p* ≤ 0.02: MUL1(−/−) *vs*. WT. **(D)** Western blot analysis to monitor the Akt1, Akt2, and Akt3 protein levels, as well as the phosphorylated AKT form (P-AKT Thr 308) and the GSK-3β substrate (P-GSK-3β S9) in WT and MUL1(−/−) cells. **(E)** Graph represents the densitometrical analysis of upregulated proteins shown in panel (D), normalized against β-actin. Results shown as means ± SD of three independent experiments. *, *p* ≤ 0.03: MUL1-(−/−) *vs*. WT.

MUL1-mediated K48 polyubiquitination invariably targets its substrates for proteasomal degradation and therefore regulates their protein level. There are four such known substrates: MFN2, ULK1, AKT, and the UBXN7 protein that controls the protein level of HIF-1α (Figure 1A) (Li et al., 2008; Bae et al., 2012; Cilenti et al., 2014; Yun et al., 2014; Li et al., 2015; Cilenti et al., 2020). Figure 1B shows the protein level of UBXN7, HIF-1α, AKT, ULK1, and MFN2 in two independent clones of HEK293 WT or MUL1(−/−), where MUL1 has been inactivated using CRISPR-Cas9 (Peng et al., 2016; Steffen et al., 2017; Puri et al., 2019; Cilenti et al., 2020). The absence of MUL1 ligase leads to the significant accumulation of all these substrate proteins (Figure 1C). AKT defines a family of three different but highly homologous kinases, Akt1, Akt2, and Akt3 (Gonzalez and McGraw, 2009). To determine which one of them is regulated by MUL1, we monitored their respective protein level in HEK293 MUL1(−/−) cells using specific antibodies. Figure 1D shows that Akt2 alone is regulated in the absence of MUL1, and there was no detectable change in either Akt1 or Akt3 protein level. In addition, the accumulation of Akt2 in HEK293 MUL1(−/−) cells leads to its activation, as seen by the degree of autophosphorylation (P-Akt2 Thr308) as well as the increased phosphorylation of its GSK-3β S9 substrate (Figures 1D,E) (Kuo et al., 2008; Le Grand et al., 2014). To verify that our results were not restricted to HEK293 MUL1(−/−) cells alone, we created a HeLa MUL1(−/−) cell line. Supplementary Figures S1A, B show that in HeLa MUL1(−/−) cells, UBXN7, HIF-1α, and Akt2 protein levels, as well as their activation reflected in the upregulation of GLUT1 protein and GSK-3β S9 phosphorylation, closely mirror the result observed in HEK293 MUL1(−/−) cells.

### Contribution of HIF-1α and Akt2 to Mitochondrial Respiration and the Glycolytic Phenotype of HEK293 MUL1(−/−) Cells

**FIGURE 2:**
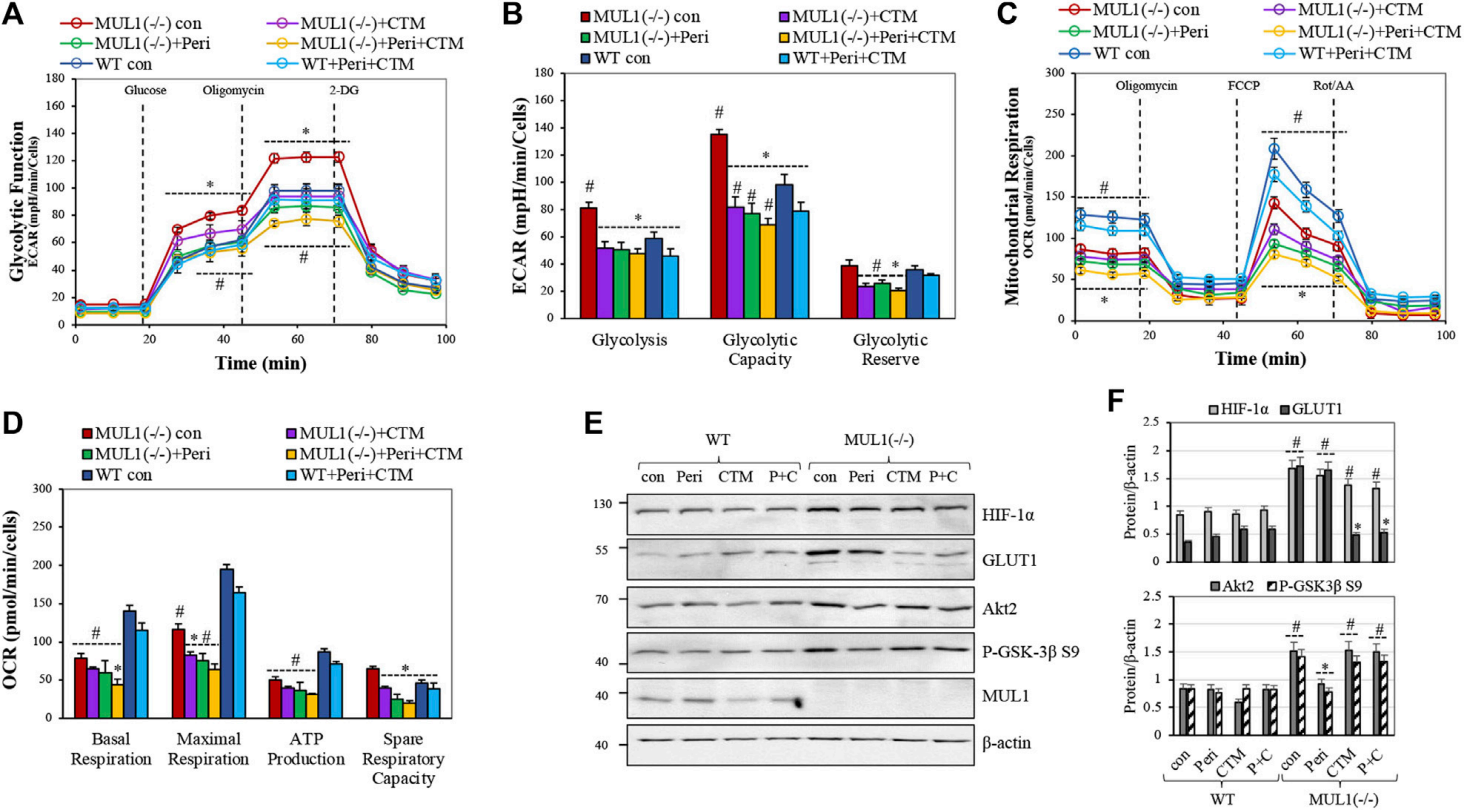
The role of Akt2 and HIF**-**1α in mitochondrial respiration and glycolytic phenotype of HEK293 MUL1(−/−) cells. HEK293 WT and MUL1(−/−) cells were treated with either 2.5 μM perifosine (Peri), 500 nM chetomin (CTM), or both drugs for 4 h. **(A)** Glycolytic capacity in WT and MUL1(−/−) cells was monitored using the glycolytic stress test. Extracellular acidification rate (ECAR) was measured using the Seahorse extracellular flux analyzer. #, *p* ≤ 0.04: MUL1(−/−) con *vs*. WT con; *, *p* ≤ 0.03: MUL1(−/−) treated *vs*. MUL1(−/−) con. **(B)** Quantification of the glycolysis, glycolytic capacity, and glycolytic reserve obtained from three independent experiments. #, *p* ≤ 0.04: MUL1(−/−) con *vs*. WT con; *, *p* ≤ 0.03 for MUL1(−/−) treated *vs*. MUL1(−/−) con. **(C)** Mitochondrial respiration in WT and MUL1(−/−) cells was monitored using the mitochondrial stress test. Oxygen consumption rate (OCR) was measured with Seahorse analyzer. **(D)** Quantification of the mitochondrial respiration data for basal respiration, maximal respiration, ATP production, and spare respiratory capacity obtained from three independent experiments. *, *p* ≤ 0.03: MUL1(−/−) treated cells *vs*. MUL1(−/−) con, #, *p* ≤ 0.04: MUL1(−/−) con *vs*. WT con. Data from three separate experiments are presented as means ± SEM. **(E)** The activity of HIF-1α and Akt2 proteins, in the presence or absence of the inhibitors, was verified by monitoring the expression of the respective target proteins, GLUT1 and P-GSK-3β S9, using western blot analysis. **(F)** Graphs represent the densitometrical analysis of the proteins shown in panel (E), normalized against β-actin. Results shown as means ± SD of three independent experiments. #, *p* ≤ 0.03 *vs*. WT con, *, *p* ≤ 0.02: MUL1(−/−) treated cells *vs*. MUL1(−/−) con.

The glycolytic stress test was performed using a Seahorse XF^e^24 analyzer. Glycolysis, glycolytic capacity, and glycolytic reserve in HEK293 WT and MUL1(−/−) cells were monitored. The individual involvement of HIF-1α and/or Akt2 proteins, in this process, was investigated using a specific HIF-1α inhibitor, chetomin, or an Akt2 inhibitor, perifosine (Kung et al., 2004; Staab et al., 2007; Li et al., 2011; Le Grand et al., 2017). Figure 2A compares the glycolytic function by the real-time measurement of the extracellular acidification rate (ECAR) in HEK293 WT and MUL1(−/−) cells. Treatment of HEK293 MUL1(−/−) cells with perifosine reduced ECAR and glycolysis to a level lower than that present in HEK293 WT cells (Figures 2A,B). Chetomin alone or in combination with perifosine works as a very potent inhibitor of the mitochondrial glycolytic function (Figure 2A). These results clearly demonstrate that both Akt2 and HIF-1α proteins work synergistically and are indispensable for the glycolytic phenotype observed in HEK293 MUL1(−/−) cells. Figure 2B represents the quantification of the measurements of glycolysis, glycolytic capacity, and the glycolytic reserve in cells untreated or treated with HIF-1α and Akt2 inhibitors. We also performed a mitochondrial respiration assay, and Figure 2C shows the trace of the oxygen consumption rate (OCR) of HEK293 WT and MUL1(−/−) cells untreated or treated with perifosine and/or chetomin. The OCR of HEK293 MUL1(−/−) is lower compared to that of the HEK293 WT cells and is further reduced after treatment with perifosine, chetomin, or with both inhibitors. Figure 2D shows the quantitative assessment of the data comparing the basal, maximal respiration, ATP production, and spare respiratory capacity. In addition, perifosine and chetomin at the concentration used here are potent inhibitors of mitochondrial respiration and they are very effective against their respective targets of HIF-1α and Akt2 (Figure 2E). Chetomin significantly reduces the expression of GLUT1, and perifosine inhibits Akt2 kinase activity (Figures 2E,F).

### Regulation of the Metabolic Flux in HEK293 Cells

**FIGURE 3:**
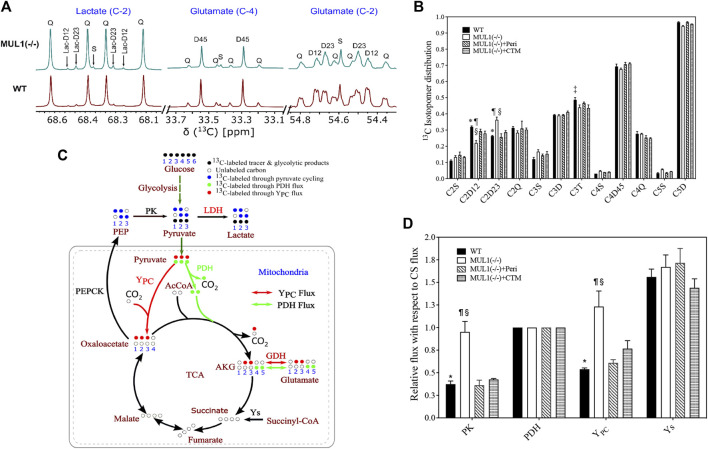
Expanded ^13^C NMR spectra of HEK293 WT and HEK293 MUL1(−/−) cells. **(A)** The labeling pattern of lactate (C-2), glutamate (C-4), and glutamate (C-2). Lactate-C2 spectra indicating that pyruvate cycled through the pyruvate kinase (PK) flux. The C2D12 and C2D23 represent the [1,2–^13^C]lactate and [2,3–^13^C]lactate isotopomers, respectively, whereas 2Q signals represent [U-^13^C]lactate. S: singlet; D12, D23, and D45: doublet; Q: quartet. **(B)** Glutamate ^13^C signal ratio obtained from ^13^C-NMR spectra of HEK293 WT, MUL1(−/−), MUL1(−/−)+Peri, and MUL1(−/−)+CTM cells utilizing the [U-^13^C]glucose. *p* ≤ 0.05: *, WT vs. MUL1(−/−); #, WT vs. MUL1(−/−)+Peri; ‡, WT vs. MUL1(−/−)+CTM; ¶, MUL1(−/−) vs. MUL1(−/−)+Peri; §, MUL1(−/−) vs. MUL1(−/−)+CTM; £, MUL1(−/−)+Peri vs. MUL1(−/−)+CTM. **(C)** Metabolic flux model demonstrating the ^13^C-labeling pattern of the metabolites derived from [U-^13^C]glucose [U-^13^C]glucose-derived [U-^13^C]pyruvate enters the TCA cycle through YPC (red dots) or PDH flux (green dots). Glucose oxidation through PDH flux labeled C4–C5 of glutamate (green dots) and C2–C3 of glutamate (red dots) through YPC flux. Key enzymatic steps involved in the metabolic flux model are as follows: (1) lactate dehydrogenase (LDH), (2) pyruvate kinase (PK), (3) pyruvate dehydrogenase (PDH), (4) pyruvate carboxylase (YPC), (5) glutamate dehydrogenase (GDH), (6) phosphoenolpyruvate carboxykinase (PEPCK), and (7) anaplerosis *via* succinyl-CoA (Ys) (note: metabolic model demonstrates the 1/2 turn of the tricarboxylic acid (TCA) cycle). **(D)** Metabolic flux rates calculated from the ^13^C-isotopomers of glutamate observed in the ^13^C-NMR spectra. Signal areas obtained from the results of the peak fitting procedure were used as an input to a metabolic model and solved numerically using tcaCALC. All flux rates are referenced to a citrate synthase (CS) flux of 1 and is equivalent to Kreb’s cycle flux. Statistical significance was *p* ≤ 0.05: *, WT vs. MUL1(−/−); #, WT vs. MUL1(−/−)+Peri; ‡, WT vs. MUL1(−/−)+CTM; ¶, MUL1(−/−) vs. MUL1(−/−)+Peri; §, MUL1(−/−) vs. MUL1(−/−)+CTM; £, MUL1(−/−)+Peri vs. MUL1(−/−)+CTM.

We investigated the effects of MUL1 inactivation and the role of HIF-1α and Akt2 accumulation on the metabolic flux using the NMR-based ^13^C-isotopomer analysis. HEK293 MUL1(−/−) treated with Akt2 or HIF-1α inhibitors and WT cells were labeled using a [U-^13^C]glucose tracer in the cell culture medium. After multiple turns of the TCA cycle, all carbon positions of the glutamate were labeled, resulting in a complex but well-defined pattern of ^13^C-NMR spectra (Purmal et al., 2014; Jin et al., 2016). A representative ^13^C-NMR spectrum of glutamate is shown in Figure 3A demonstrating the ^13^C-labeling pattern of C-2 and C-4 carbons. The results of the glutamate ^13^C signal ratio obtained from ^13^C-NMR spectra of HEK293 WT, MUL1(−/−), MUL1(−/−) + perifosine, and MUL1(−/−) + chetomin are summarized in Figure 3B. The schematic diagram of the metabolic flux model in Figure 3C shows the ^13^C-isotope labeling patterns of the metabolites derived from the [U-^13^C]glucose metabolism after a single turn of the cycle [U-^13^C]glucose-derived [U-^13^C]pyruvate produces lactate *via* LDH and can enter the tricarboxylic acid (TCA) cycle through Y_PC_ (red arrow) and PDH flux (green arrow). In the first turn of the TCA cycle, the [U-^13^C]pyruvate oxidation through PDH produces labeled glutamate C4–C5 (green dots) positions, whereas Y_PC_ flux labels glutamate C2–C3 (red dots). Pyruvate cycling *via* phosphoenolpyruvate carboxykinase (PEPCK) and PK fluxes is also shown in Figure 3C. Pyruvate cycling was included as a possible pathway, as the presence of [1,2–^13^C_2_] and [2,3–^13^C_2_]lactate isotopomers is only possible via this pathway (Figure 3A). The relative metabolic flux rates of PK, Y_PC_, pyruvate dehydrogenase (PDH), and anaplerosis leading to succinyl-CoA (Y_s_) were estimated using glutamate ^13^C-isotopomer distribution data obtained from ^13^C-NMR analysis of the cell extracts. All flux rates are referenced to a citrate synthase (CS) flux of 1 and is equivalent to Kreb’s cycle flux. Using tcaCALC, multiple different metabolic models can be compared to each other, with the most parsimonious model selected (Figure 3D) (Alger et al., 2021). HEK293 MUL1(−/−) cells significantly increased the PK and Y_PC_ fluxes compared to WT cells, and the Akt2 or HIF-1α inhibitors significantly decreased the PK and Y_PC_ flux rates in MUL1(−/−) cells compared to untreated cells (Figure 3D). Additionally, we have performed the same metabolic flux analysis using the [U-13C]glucose tracer for HeLa WT and HeLa MUL1(−/−) cells to confirm the effect of MUL1 knockout on energy metabolism in an alternative cell line. Glutamate isotopomer analysis of HeLa WT and MUL1(−/−) cells shows the same effects of increased pyruvate anaplerosis into the TCA cycle as well as he significant flux through PK (Supplementary Figures S1C, D). These results closely mirror those seen in the HEK293 cells.

### Effect of HIF-1α Activation in the Regulation of Glycolysis in HEK293 Akt2(−/−)

**FIGURE 4:**
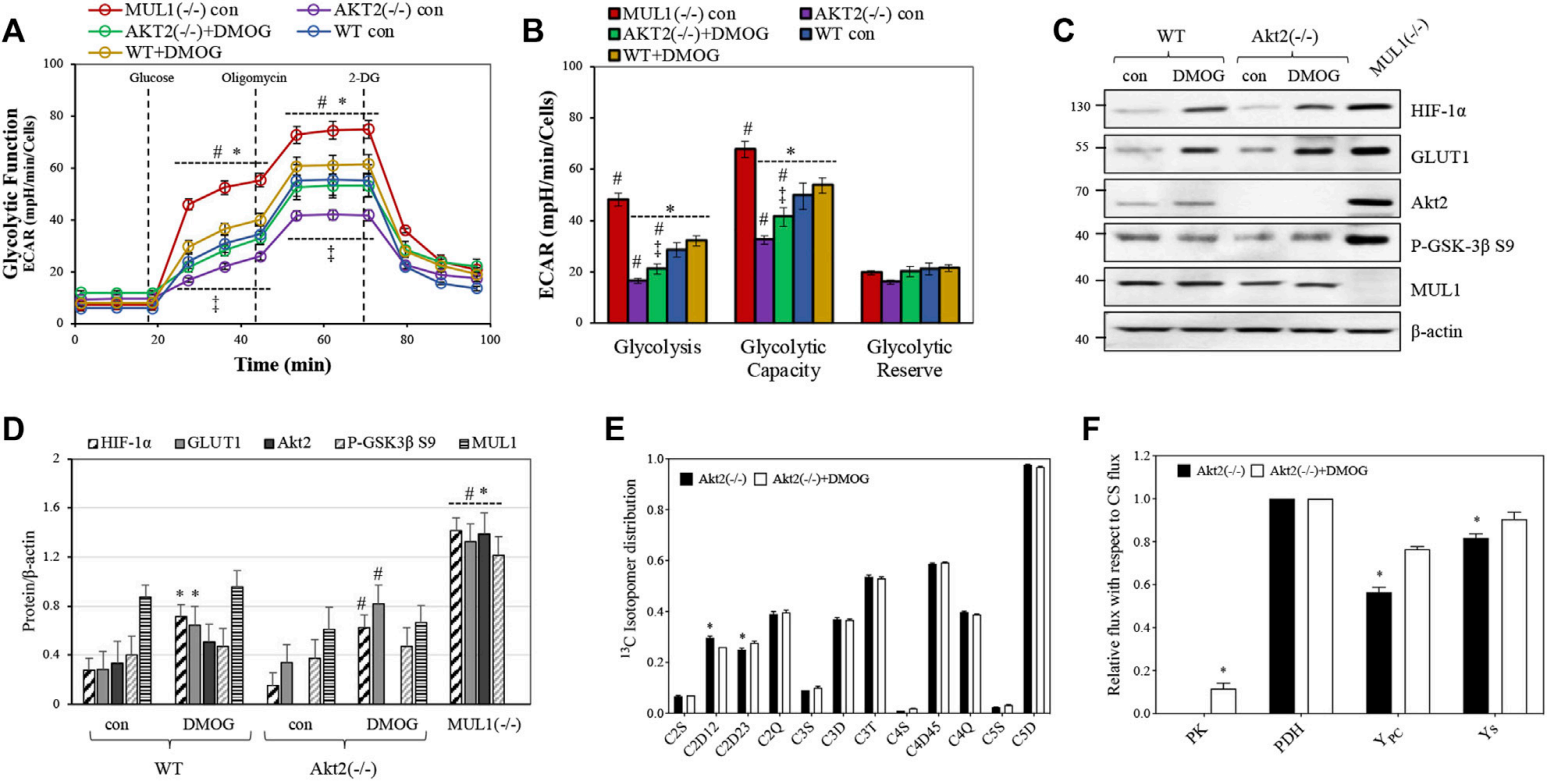
Akt2 and HIF-1α proteins contribute to support the metabolic phenotype of HEK293 MUL1(−/−) cells. **(A)** Glycolytic capacity in HEK293 WT and Akt2(−/−) cells before or after treatment with the HIF-1α activator DMOG (100 μM for 4 h). Extracellular acidification rate (ECAR) was measured using the Seahorse analyzer. HEK293 MUL1(−/−) cells were used as a positive control. #, *p* ≤ 0.035: *vs*. WT con*;* *, *p* ≤ 0.035: *vs*. MUL1(−/−) con; ‡, *p* ≤ 0.045: AKT2(−/−) con vs. AKT2(−/−)+DMOG. **(B)** Quantification of the glycolysis, glycolytic capacity, and glycolytic reserve obtained from three independent experiments. Data from three separate experiments are presented as means ± SEM. #, *p* ≤ 0.03: *vs*. WT con*;* *, *p* ≤ 0.035: *vs*. MUL1(−/−) con; ‡, *p* ≤ 0.045: AKT2(−/−) con vs. AKT2(−/−)+DMOG. **(C)** The effect of HIF-1α activator, DMOG, on the expression levels of HIF-1α, GLUT1, Akt2, P-GSK-3β S9, in Akt2(−/−), or MUL1(−/−) cells was monitored by western blot analysis. **(D)** Densitometrical analysis of the proteins shown in panel (C), normalized against β-actin. Results shown as means ± SD of three independent experiments. *, *p* ≤ 0.03 *vs*. WT con; #, *p* ≤ 0.025 *vs*. Akt2(−/−) con. **(E)** Glutamate ^13^C signal ratio obtained from ^13^C-NMR spectra of Akt2(−/−) and Akt2(−/−) +DMOG cells utilizing the [U-^13^C]glucose (note: S, D, T, and Q are singlet, doublet, triplet, and quartet, respectively. All signal ratios were calculated with respect to the total area of the corresponding glutamate resonance. Data are represented as mean ± SEM. **(F)** Metabolic flux rates calculated from the ^13^C-isotopomers of glutamate observed in the ^13^C-NMR spectra. Signal areas obtained from the results of the peak fitting procedure were used as an input to a metabolic model and solved numerically using tcaCALC. All flux rates are referenced to a citrate synthase (CS) flux of 1 and is equivalent to Kreb’s cycle flux. Statistical significance was *p* ≤ 0.05: *, for Akt2(−/−) vs. Akt2(−/−)+DMOG.

We created HEK293 Akt2(−/−) cells using CRISPR-Cas9 (see Methods) in order to investigate the role of HIF-1α in glycolysis in the absence of the Akt2 protein. ECAR was measured for both HEK293 Akt2(−/−) and WT cells in the presence or absence of dimethyloxalyglycine (DMOG), a known activator of HIF-1α (Zhdanov et al., 2015). HEK293 Akt2(−/−) had substantially reduced glycolysis compared to MUL1(−/−) cells (Figure 4A). When HIF-1α was activated in HEK293 Akt2(−/−) cells, there was a small but significant increase in glycolysis, as well as glycolytic capacity (Figure 4B). The same treatment in HEK293 WT cells was unremarkable (Figures 4A,B). Figure 4C shows the western blot analysis to monitor the protein expression and activity of HIF-1α and Akt2 proteins in HEK293 WT and Akt2(−/−) cells before or after treatment with DMOG. Figure 4D shows a densitometric analysis of the western blot data from 4C normalized against β-actin. The combined data from these experiments strongly suggest that the accumulation and activation of both Akt2 and HIF-1α are required for the glycolytic phenotype observed in MUL1(−/−) cells. Furthermore, we performed a^13^C-NMR-based metabolic flux analysis to determine the effect of HIF-1α on metabolism in HEK293 cells by HIF-1α activation in the absence of Akt2 protein. The isotopomer results from ^13^C-NMR spectra of HEK293 Akt2(−/−) and Akt2(−/−) + DMOG cells are summarized in Figure 4E. The ^13^C-labeling pattern in glutamate for C2D12 and C2D23 multiplets was significantly different between HEK293 Akt2(−/−) and Akt2(−/−) + DMOG cells (Figure 4E). Metabolic flux analysis results suggested that the activation of HIF-1α by DMOG significantly increased PK, Y_PC_, and Y_S_ fluxes (Figure 4F). The metabolic fluxes of Akt2(−/−) cells with or without DMOG were also compared with those of HEK293 WT and MUL1(−/−) cells and are plotted in Supplementary Figure S2. Akt2 knockout significantly downregulates the PK flux compared to HEK293 WT cells. PK flux was partially restored with DMOG but did not reach the WT levels, while Y_PC_ was elevated above WT but fell short of reaching MUL1(−/−) levels (Supplementary Figure S2). Anaplerosis via succinyl-CoA (Y_s_) was significantly lower in both Akt2(−/−) and Akt2(−/−) + DMOG cells compared to that of WT and MUL1(−/−) cells (Supplementary Figure S2). All flux rates are referenced to a citrate synthase (CS) flux of 1 and is equivalent to Kreb’s cycle flux.

### The Role of MUL1 in the Metabolic Homeostasis

**FIGURE 5:**
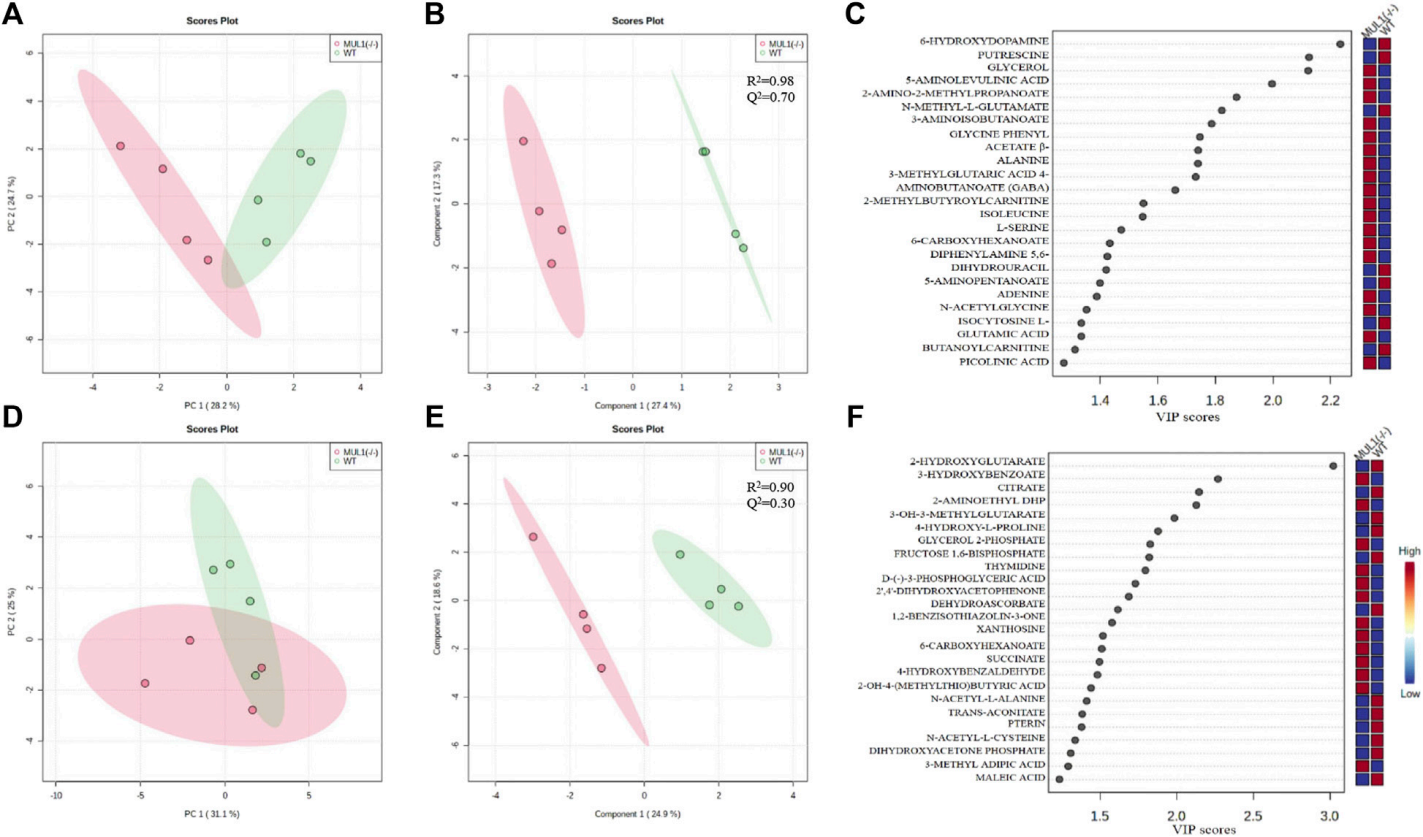
Metabolomic comparison of HEK293 WT and HEK393 MUL1(−/−) cells. **(A)** 2D score plot of the principal component analysis (PCA) from LC-MS positive ion mode data. The degree of variance is displayed in parentheses on each axis, and the shaded areas indicate the 95% confidence regions based on the data points for each group. **(B)** 2D score plot of the partial least-squares discriminant analysis (PLS-DA) from LC-MS positive ion mode data. The amount of variance explained is displayed in parentheses on each axis, and the shaded areas indicate the 95% confidence regions based on the data points for each groups in PLS-DA models. Supervised PLS-DA maximizes the separation between both groups using the group label, and cross-validation was also used to determine the optimal number of components required to build the PLS-DA model. The sum of squares captured by the model (*R* ^2^) and the cross-validated *R* ^2^ (Q^2^) parameters were used to determine the robustness of the mathematical model. **(C)** Corresponding variable importance in projection (VIP) score plot of the PLS-DA model B. Variable importance in projection (VIP) score plots from the PLS-DA model demonstrating the differences in the level of top 25 metabolites between HEK293 WT and MUL1(−/−) cells. **(D)** 2D score plot of PCA from LC-MS negative ion mode data. **(E)** 2D score plot of the PLS-DA model from the LC-MS negative ion mode data. **(F)** PLS-DA variable importance in projection (VIP) score plot of the PLS-DA model E.

We performed detailed global metabolic studies using HEK293 MUL1(−/−) and WT cells. The unsupervised principal component analysis (PCA) 2D score plots showed a clear separation in the metabolic profile between HEK293 WT and MUL1(−/−) cells in the LC-MS positive ion mode (Figure 5A), whereas for the LC-MS negative ion mode data, partial separation was achieved between both groups in PCA (Figure 5D). The supervised partial least-squares discriminant analysis (PLS-DA) model was used to predict the classes of samples and maximize the separation between groups (Figures 5B–E). To determine the robustness of the mathematical model, *R* ^2^ and Q^2^ parameter values were calculated for the PLS-DA model. The *R* ^2^/Q^2^ values for the LC-MS positive and negative ion mode data were 0.98/0.70 and 0.90/0.30, respectively (Figures 5B–E). The PLS-DA variable importance projection (VIP) score plot from the LC-MS positive and negative mode PLS-DA models identified a large group of metabolites different from HEK293 WT and MUL1(−/−) cells (Figures 5C–F). Heatmaps of the top 25 metabolites from both positive and negative mode MS demonstrated excellent clustering of the samples based on MUL1 status (Supplementary Figures S3A, B). Significantly different metabolites were identified by either PLS-DA or *t*-test, class membership for each sample. The semi-quantitative metabolomic panel from the LC-MS positive and negative mode data in Supplementary Figure S4 summarized the significantly different metabolites between HEK293 WT and MUL1(−/−) cells. The metabolites such as β-alanine, glycerol, glycine, N-acetylglycine, serine, succinate, glutamic acid, isoleucine, thymidine, GABA, and picolinic acid were found to be significantly higher in HEK293 MUL1(−/−) cells, whereas N-acetyl-alanine, citrate, trans-aconitate, 3-methylglutaric acid, proline, 2-hydroxybutyric acid, and 5-amino pentanoate were significantly higher in HEK293 WT cells (Supplementary Figure S4). All significantly different metabolites between HEK293 WT and MUL1(−/−) cells were utilized for metabolite set enrichment analysis (MSEA) to produce a network of pathways. MSEA results indicate that the provided metabolite set has significantly enriched the glutamate, arginine, proline, and ammonia recycling metabolic pathways. In addition, glycine, serine, alanine, and propanoate metabolic pathways were also substantially enhanced in the absence of MUL1 (Supplementary Figures S5A–C).

### Alterations in Global Gene Expression and Integrated Multi-Omics Based on the Metabolic Pathways in HEK293 MUL1(−/−) Cells

**FIGURE 6:**
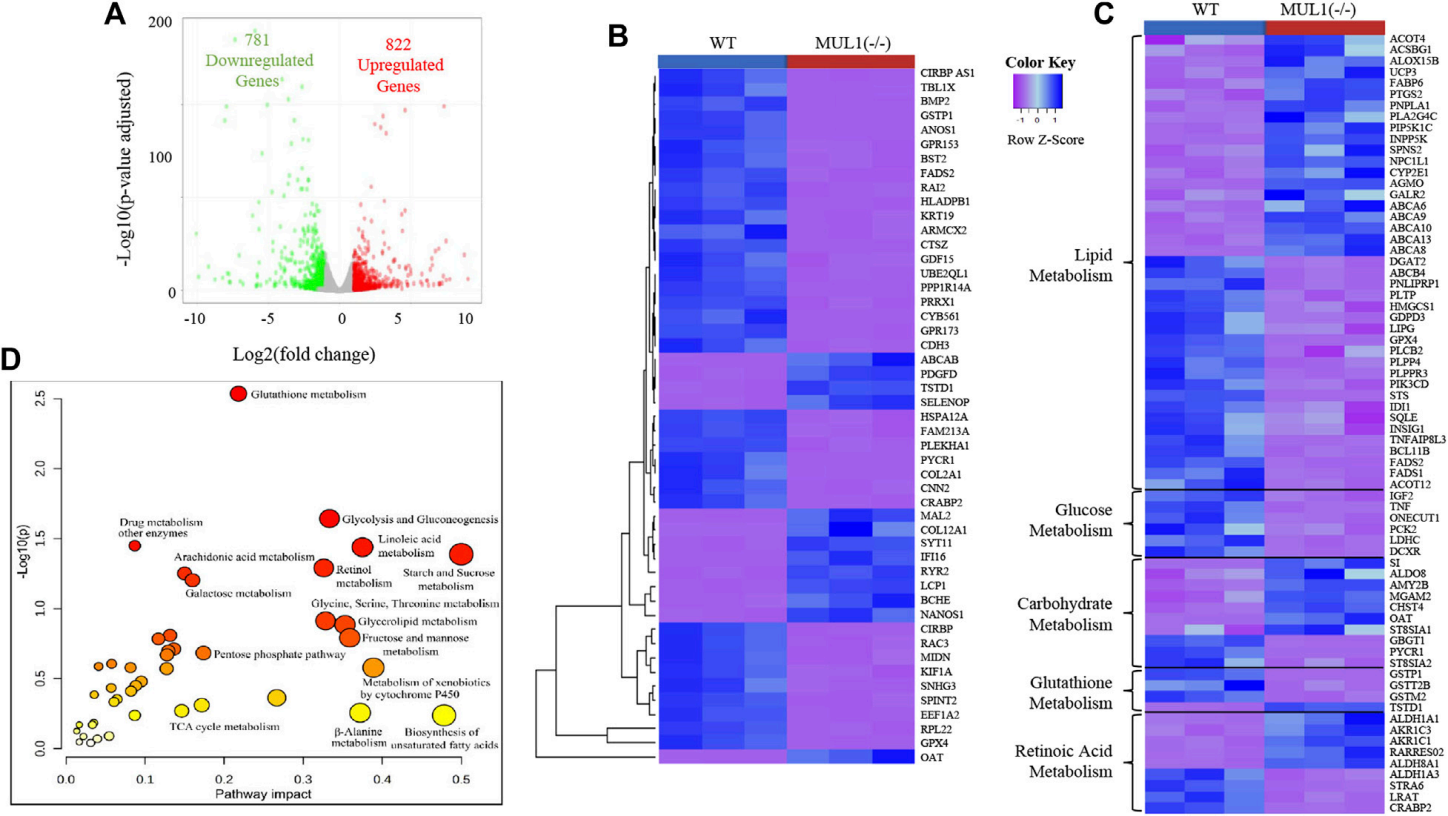
RNA sequencing and joint pathway analysis in HEK293 MUL1(−/−). **(A)** The global transcriptional change across the groups compared was visualized by a volcano plot. Each data point in the scatter plot represents a gene. The log2 fold change of each gene is represented on the *x*-axis, and the log10 of its adjusted *p*-value is on the *y*-axis. Genes with an adjusted *p*-value less than 0.05 (*p < 0.05)* and a log2 fold change greater than 1 are indicated by red dots. These represent the upregulated genes. Genes with an adjusted *p < 0.05* and a log2 fold change less than -1 are indicated by green dots. These represent the downregulated genes. **(B)** Heatmap of top 50 differentially expressed genes shown for HE293 WT and MUL1(−/−) cells. **(C)** Heatmap of the differentially expressed genes involved in cellular metabolism. Most of the genes related to the glucose and glutathione metabolism are downregulated in HEK293 MUL1(−/−) cells. The genes related to fatty acid and retinoic acid metabolism are upregulated and downregulated in HEK293 MUL1(−/−) cells. **(D)** The fold change in HEK293 MUL1(−/−) cells versus the WT control was used as an input to perform the joint metabolic pathway analysis employing the gene and metabolite from uniport proteins ID and HMDB ID, respectively. Metabolic pathways containing both metabolites and metabolic genes were used for the integrative pathway analysis along with Fisher’s exact test for the enrichment analysis. The metabolic pathways with higher *p-*values and pathway impacts were significantly altered in HEK293 MUL1(−/−) cells.

Genome-wide RNA sequencing data comparing HEK293 WT and MUL1(−/−) gene expression showed that 781 genes were downregulated, whereas 822 genes were upregulated in MUL1(−/−) cells (Figure 6A). The heatmap of the top 50 differentially expressed genes shows excellent clustering and classification between both groups (Figure 6B). The functional enrichment analysis of data identified that a large proportion of the regulated genes in HEK293 MUL1(−/−) cells are important for metabolic processes, including lipid, glucose, retinoic acid, carbohydrate, and glutathione metabolism. Our analysis indicated that the deletion of MUL1 upregulates a group of genes including CYP2E1, FABP6, PNPLA1, ACSBG1, AKR1C3, AKR1C1, and PIP5K1C that are involved in lipid transport, prostaglandin, progesterone, triglyceride, and retinoic acid metabolic process. In addition, GPX4, PLCB2, PLPP4, PLPPR3, FADS1, FADS2, GSTP1, and GSTM2, genes involved in phospholipid and linoleic acid metabolic processes were downregulated. Genes involved in glucose and pyruvate metabolism (IGF2, TNF, DCXR, ONECUT1, LDHC, and PCK2) were also downregulated. A heatmap highlighting genes that are involved in the lipid, glucose, carbohydrate, glutathione, and retinoic acid metabolism is shown in Figure 6C. To take advantage of multi-omics datasets of HEK293 cells, we conducted a joint pathway analysis, employing the fold change values of the metabolites and genes in HEK293 MUL1(−/−) cells as compared to WT control cells. The results from the joint pathway analysis depicted in Figure 6D indicate that glutathione metabolism, starch and sucrose metabolism, fatty acid (linoleic acid) metabolism, glycolysis and gluconeogenesis, glycine, serine, and threonine metabolism, glycolipid metabolism, and retinol metabolism were significantly interrupted in HEK293 MUL1(−/−) cells.

### Perturbation in the Lipid Profile of HEK293 MUL1(−/−) Cells

**FIGURE 7:**
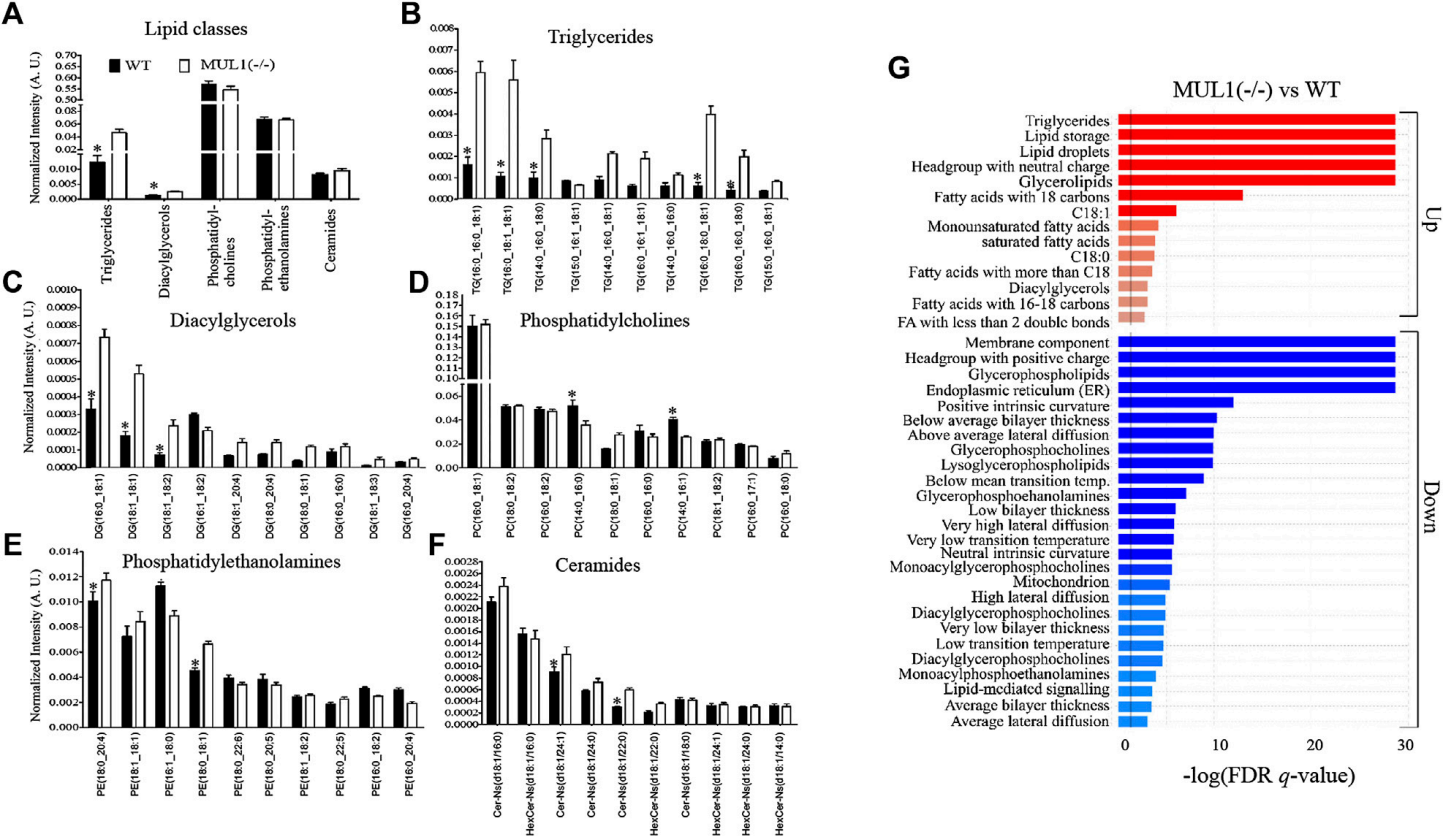
Lipidomic comparison of HEK293 WT and HEK293 MUL1(−/−) cells. **(A)** High-resolution LC-MS/MS-based lipidomic profiling of lipid classes, i.e., triglycerides (TGs), diacylglycerols (DGs), phosphatidylcholines (PCs), phosphatidylethanolamines (PEs), and ceramides in the HEK293 WT and MUL1(−/−) cells demonstrating significant changes in TG and DG lipid classes. Lipidomic profiling of 10 highest abundant **(B)** TGs, **(C)** DGs, **(D)** PCs, **(E)** Pes, and **(F)** ceramides in WT and MUL1(−/−) cells, providing more insights into the changes in the level of individual lipids. HEK293 MUL1(−/−) cells demonstrate significant changes in the level of several lipids with respect to the WT control cells (note: values for each bar are expressed as mean ± SEM (n = 4) and individual lipid intensity was normalized to the total lipid intensity of each sample. *p* ≤ 0.05: a: WT *vs*. MUL1(−/−). **(G)** Lipid ontology enrichment analysis of the MUL1(−/−) cells. Enrichment analysis of MUL1(−/−) *vs*. WT cells in the “ranking mode”. The gray vertical lines indicate the cut-off value of significantly up (red bars) and down (blue bars) lipids in MUL1(−/−) cells with respect to WT (*q <* 0.05). The bar colors are scaled with the enrichment (−log FDR *q*-values). Lipid ontology (LION): a web-based interface was used for the identification of lipid-associated terms in lipidomes.

To monitor changes in the lipid profile, LC-MS analysis was performed on HEK293 WT and MUL1(−/−) cells. The profiling of various lipid classes such as triglycerides (TGs), diacylglycerols (DGs), phosphatidylcholines (PCs), phosphatidylethanolamines (PEs), and ceramides shows strong changes in lipid metabolism enforced by the MUL1 knockout. The lipidomic profiling data demonstrated that the TGs and DGs are significantly higher in HEK293 MUL1(−/−) cells but there was no notable difference found for PCs, PEs, and ceramides (Figure 7A). To provide more insight into the lipid profiling of each lipid class, the 10 most abundant TGs, DGs, PCs, PEs, and ceramides were analyzed. TGs such as TG (16:0_16:0_18:1), TG (16:0_18:1_18:1), TG (14:0_16:0_18:0), TG (16:0_18:0_18:1), and TG (16:0_16:0_18:1), as well as DGs species, i.e., DG (16:0_18:1), DG (18:1_18:1) and DG (18:1_18:2), were found to be significantly higher in MUL1(−/−) (Figures 7B,C). PC(14:0_16:0), PC(14:0_16:1), PE (18:0_20:4), and PE (18:0_18:1) were significantly lower in MUL1(−/−) cells, whereas, PE (16:1_18:0) was found to be higher in WT cells. Ceramide species such as CerNS(d18:1/24:1) and CerNS(d18:1/20:0) were significantly higher in MUL1(−/−) cells (Figure 7F). Lipid ontology (LION) enrichment analysis was carried out for the annotation of lipids in untargeted lipidomic analysis. The results shown in Figure 7G suggest that the most important components such as lipid storage, triglycerides, headgroup with a neutral charge, glycerolipids, fatty acids with 18 carbons, and C18:1 chain length were significantly upregulated in MUL1(−/−) cells. Lipids of membrane component, headgroup with a positive charge, glycerophospholipids, lipids of endoplasmic reticulum, lysoglycerophospholipids, glycerophosphocholines, and glycerophosphoethanolamines were significantly lower in HEK293 MUL1(−/−) cells.

## Discussion

**FIGURE 8:**
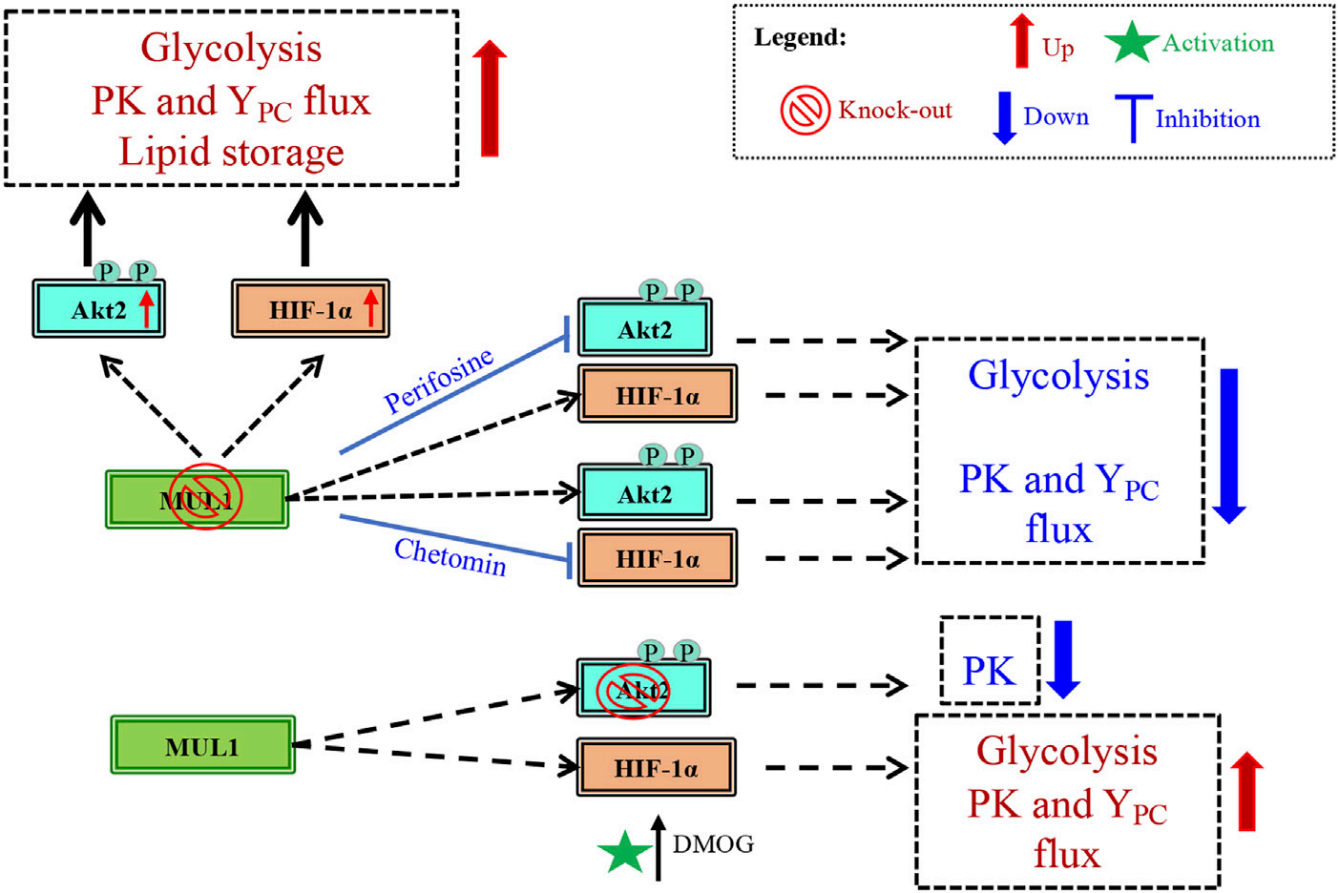
Schematic diagram of the potential MUL1-dependent pathway in the regulation of metabolism. The metabolism in MUL1(−/−) cells can be summarized as an increased glycolytic rate, increased anaplerotic (Y_PC_) and PK fluxes, and an increase in the lipid storage. This regulation in metabolism is mediated through Akt2 and HIF-1α proteins as suggested by the inhibition of these proteins, using perifosine and chetomin, respectively. The individual roles of Akt2 and HIF-1α are established in Akt2(−/−) cells resulting in reduced PK flux, whereas the activation of HIF-1α by DMOG increases glycolysis, as well as PK and Y_PC_ fluxes compared to Akt2(−/−) cells.

Metabolic reprogramming is the hallmark of cancer, but healthy mammalian cells can also modulate their metabolic state in response to fast growth demand or due to various conditions that adversely affect oxidative phosphorylation (OXPHOS), such as hypoxia (de Padua et al., 2017; Faubert et al., 2020; Sakaguchi and Kimura, 2021). Metabolic regulation involves the coordinate function of numerous proteins located in different subcellular compartments including mitochondria, cytoplasm, and nucleus. Mitochondria continuously communicate their current bioenergetic and biosynthetic states to the rest of the cell through various signaling pathways and they can alter their function to accommodate changing metabolic demands (Mishra and Chan, 2016). One of the many ways by which mitochondria communicate is through signaling proteins located in the outer mitochondrial membrane (OMM). In this present study, we focused on one such protein, the mitochondrial MUL1 E3 ubiquitin ligase and its potential role in the regulation of metabolism. MUL1 is located in the OMM, and its function has been implicated in mitophagy, cell death, mitochondrial dynamics, and innate immune response (Zhang et al., 2008; Jenkins et al., 2013; Yun et al., 2014; Peng et al., 2016; Ni et al., 2017; Puri et al., 2019; Cilenti et al., 2020). As an E3 ubiquitin ligase, MUL1 interacts with various E2 ubiquitin-conjugating enzymes to ubiquitinate specific substrates (Ambivero et al., 2014). MUL1 can perform two types of ubiquitination, where either the lysine 48 (K48) or the lysine 63 (K63) of the ubiquitin is involved in the isopeptide linkage (Glickman and Ciechanover, 2002; Livnat-Levanon and Glickman, 2011). In addition, MUL1 can function as a SUMO E3 ligase to attach SUMO (small ubiquitin-like modifier) onto specific substrates (Braschi et al., 2009; Prudent et al., 2015; Doiron et al., 2017). We investigated MUL1’s K48 ubiquitination that invariably targets substrates for proteasomal degradation. We describe that at least four substrates are regulated and accumulate in MUL1(−/−) cells: ULK1, MFN2, HIF-1α, and Akt2 proteins (see Figure 1) (Bae et al., 2012; Li et al., 2015; Escobar-Henriques and Joaquim, 2019; Cilenti et al., 2020). We focused our studies on Akt2 and HIF-1α coregulation by MUL1 since activation of these proteins is involved in metabolic phenotypes that favors glycolysis, a hallmark of cancer cells referred to as the Warburg effect (Lu et al., 2002; Pavlides et al., 2009). We found that inactivation of MUL1 leads to suppression of OXPHOS and increased glycolysis. In addition, steady-state flux rates show an increased activity of Y_PC_ and PK flux in MUL1(−/−) cells. These metabolic changes can be reversed by specific Akt2 or HIF-1α inhibitors. Furthermore, our data indicate that the metabolic phenotype of MUL1(−/−) cells is distinct from the glycolytic state observed in cancer cells (Warburg effect), where increased pyruvate dehydrogenase (PDH) and lactate dehydrogenase (LDH) switch OXPHOS to glycolysis (Park et al., 2018; Li et al., 2019). We performed detailed metabolomic analyses of MUL1(−/−) cells that clearly show that MUL1 has a very important role in both metabolic and lipidomic regulation. Detailed metabolomic and genomic analyses using HEK293 WT and MUL1(−/−) cells were employed to investigate the state of metabolic pathways. The results demonstrate that there are significant differences in the metabolites and the gene expression profiles between these 2 cell lines. The multi-omics approach identified a number of metabolic pathways that were perturbed in the absence of MUL1. These include glutathione metabolism, starch and sucrose metabolism, fatty acid metabolism, glycolysis, glycine, serine, and threonine metabolism, glycolipid metabolism, and retinol metabolism. Lipidomic analysis showed significant accumulation of neutral head groups containing lipids such as triglycerides and diacylglycerides in MUL1(−/−) cells. The overall levels of other lipid species such as phosphatidylcholines, phosphatidylethanolamines, and ceramides were unaffected by MUL1 inactivation in HEK293 cells. Figure 8 summarizes the proposed MUL1’s function and pathway in the regulation of metabolism based on the data presented here.

Our multifaceted analysis shows that MUL1 is involved in the regulation of metabolism and its inactivation/downregulation can lead to a distinct metabolic state not previously described. The MUL1 protein level is known to be regulated, but the mechanism(s) is not fully characterized. Our previous studies identified Omi/HtrA2, a serine protease located in the mitochondrial intermembrane space (IMS), as a major regulator of the MUL1 protein level (Cilenti et al., 2014). Omi/HtrA2 has a role in protein quality control within the IMS, and its activity is modulated by oxidative stress, the HAX1 protein, as well as by the NDUFA13 subunit of mitochondrial respiratory chain complex I (Faccio et al., 2000; Cilenti et al., 2004; Liu et al., 2010; Chaganti et al., 2013; Ruan et al., 2017; Kummari et al., 2021). In addition, K48-autoubiquitination of MUL1 is another potential mechanism that can target the ligase for degradation and regulate its protein level (Li et al., 2015; Kim et al., 2018).

Besides the involvement of Akt2 and HIF-1α in the regulation of metabolism by MUL1, the participation of other MUL1-substrate proteins, such as the MFN2 and ULK1 proteins, cannot be excluded. Accumulation of MFN2 and ULK1 is also observed in MUL1(−/−) cells. In addition, MFN2 has been implicated in the regulation of metabolism in cancer cells and shown to interact with PKM2 (Nemazanyy et al., 2013; Li et al., 2019). ULK1 plays a major role in autophagy but can also phosphorylate key glycolytic enzymes, such as hexokinase (HK), phosphofructokinase 1 (PFK1), enolase 1 (ENO1), and fructose-1,6-bisphosphatase (FBP1), in response to nutritional deprivation (Li et al., 2016). This dual parallel function of ULK1 can sustain the activity of multiple glycolytic enzymes and support metabolic homeostasis during amino acid and growth factor deprivation (Li et al., 2016). In our studies, we used specific inhibitors to establish that Akt2 and HIF-1α are the main “drivers” of the MUL1(−/−) metabolic phenotype; any potential role MFN2 and/or ULK1 might have in this process will be downstream of these two proteins.

## Conclusion

We identified a new function for the mitochondrial MUL1 E3 ubiquitin ligase in the regulation of metabolism. The mechanism involves the K48-polyubiquitinating function of the ligase and the coregulation of Akt2 and HIF-1α proteins. The accumulation and co-activation of Akt2 and HIF-1α in MUL1(−/−) cells drive and maintain this new metabolic phenotype characterized by the activated pyruvate carboxylation and PK flux, along with increased aerobic glycolysis. In addition, MUL1(−/−) cells have a distinct lipid metabolism characterized by increased triglyceride and diacylglycerol storage. These results support a very important role for MUL1 ligase in the regulation of mitochondrial metabolism and lipogenesis including a new metabolic state of aerobic glycolysis.

## Data Availability

The metabolite information was submitted to MetaboLights public repository (https://www.ebi.ac.uk/metabolights/editor/guide/upload/MTBLS4729). The mRNA sequence data were submitted to GEO archive https://www.ncbi.nlm.nih.gov/geo/subs/, accession number GSE201725.
