# Supplementary material for: Regulation of Metabolism by Mitochondrial MUL1 E3 Ubiquitin Ligase
Source: Front Cell Dev Biol. 2022 Jun 29;10:904728. doi: 10.3389/fcell.2022.904728 (PMC9277447; doi:10.3389/fcell.2022.904728)
Supplement: Supplementary file 1 [file DataSheet1.PDF]

Supplementary data

Supplementary Figure S1

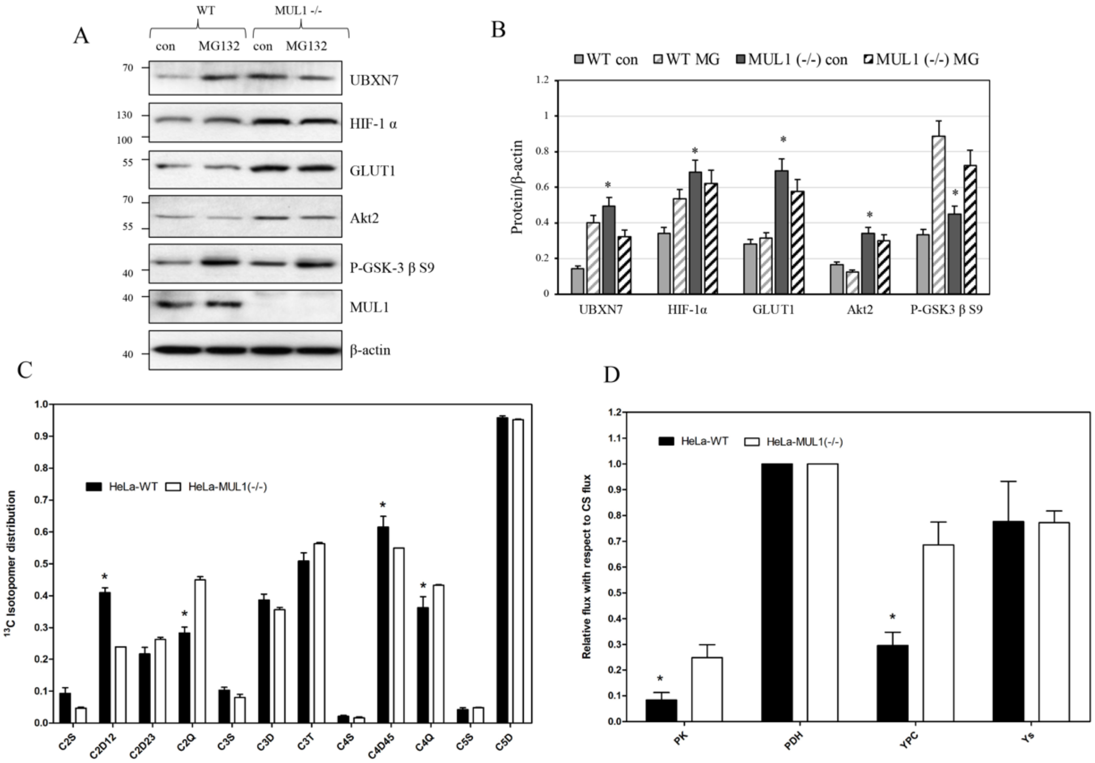

Figure S1. MUL1 inactivation in HeLa cells affects Akt2 and HIF-1α, protein levels and metabolic flux. (A) HeLa WT and HeLa MUL1(-/-) cells were treated with 5 μM of MG132 for 4 hours; whole cell extracts were prepared, and Western blot analysis was used to monitor UBXN7, HIF-1α, GLUT1, Akt2, P-GSK-3β S9, as well as MUL1 protein expression. β-actin was used to verify equal loading in each lane. **(B)** Graph represents the densitometric analysis of the proteins shown in **A**, normalized against β-actin. Results shown as means ± SD of three independent experiments. *, $P < 0.04$ MUL1(-/-) con *vs* WT con. (**C**) Glutamate ^{13}C signal ratio obtained from ^{13}C-NMR spectra of HeLa WT and HeLa MUL1(-/-) cells utilizing the [U-^{13}C]glucose All signal ratio were calculated with respect to the total area of the corresponding glutamate resonance. (**D**) Steady-state flux rates, relative to a Kreb's cycle flux, are calculated from the isotopomers of glutamate observed at carbon positions 2, 3, 4 and 5 in the ^{13}C NMR spectra and modeled using tcaCALC. All flux rates are referenced to a citrate synthase (CS) flux of 1 and is equivalent to a Kreb's cycle flux. Pyruvate dehydrogenase (PDH), pyruvate carboxylase (YPC), pyruvate kinase (PK), and anaplerosis leading to succinyl-CoA (YS). $P \leq 0.05$ and indicated by *, HeLa WT vs MUL1(-/-) cells. S, D, T, and Q are singlet, doublet, triplet and quartet, respectively. Data is represented as mean ±SEM.

Supplementary Figure S2

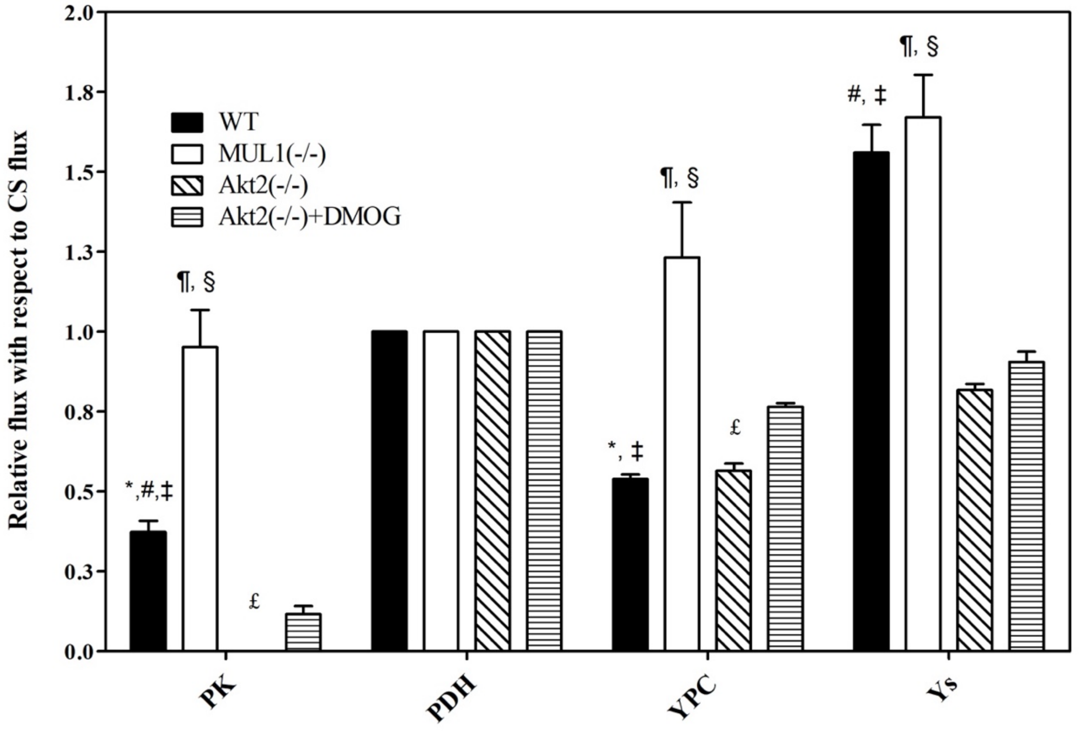

Figure S2. Metabolic flux rates derived from the ^{13}C-isotopomers of glutamate observed in the ^{13}C-NMR spectra. Signal areas were used as an input to derive metabolic model and solved numerically using tcaCALC. All flux rates are referenced to a citrate synthase (CS) flux of 1 and is equivalent to a Kreb's cycle flux. Statistical significance was $P \leq 0.05$: *, WT vs MUL1(-/-); #, WT vs Akt2(-/-); ‡, WT vs Akt2(-/-)+DMOG; ¶, MUL1(-/-) vs Akt2(-/-); §, MUL1(-/-) vs Akt2(-/-)+DMOG, and £, Akt2(-/-) vs Akt2(-/-)+DMOG.

Supplementary Figure S3

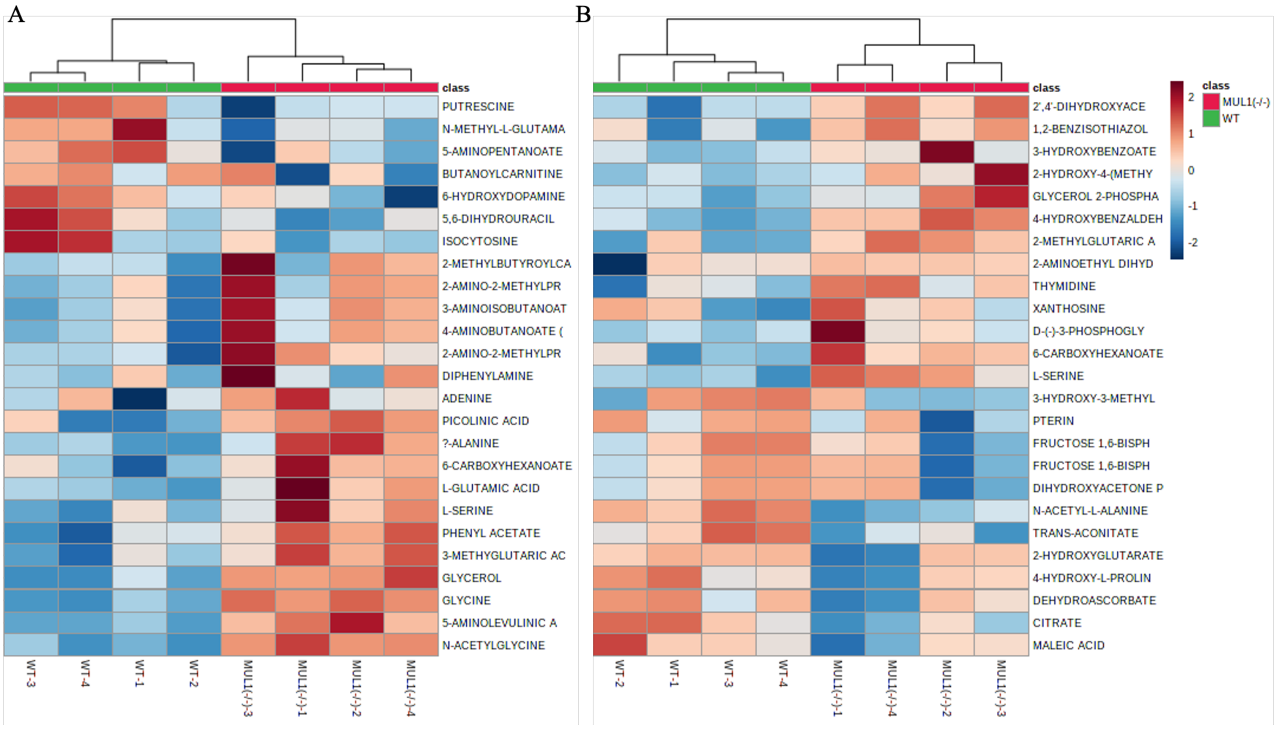

Figure S3. Clustering of the top twenty-five metabolites between HEK293 WT and MUL1(-/-) cells. (A) Clustering result shown as heatmap and demonstrating the differences in the level of the top 25 metabolites between HEK293 WT and MUL1(-/-) cells identified *via* PLS-DA and VIP scores, using LC-MS data in the positive mode. (**B)** Clustering result shown as heatmap that demonstrate the differences in the level of the top 25 metabolites between HEK293 WT and MUL1(-/-) cells identified *via* PLS-DA and VIP scores, using LC-MS data in the negative mode. (Note: The scale bar represents normalized intensities of the features. Heatmap visualizes a numerical table into a corresponding 2D color map to provide an overview of the data values, indicating level changes from low (cold) to high (hot) intensity.)

Supplementary Figure S4

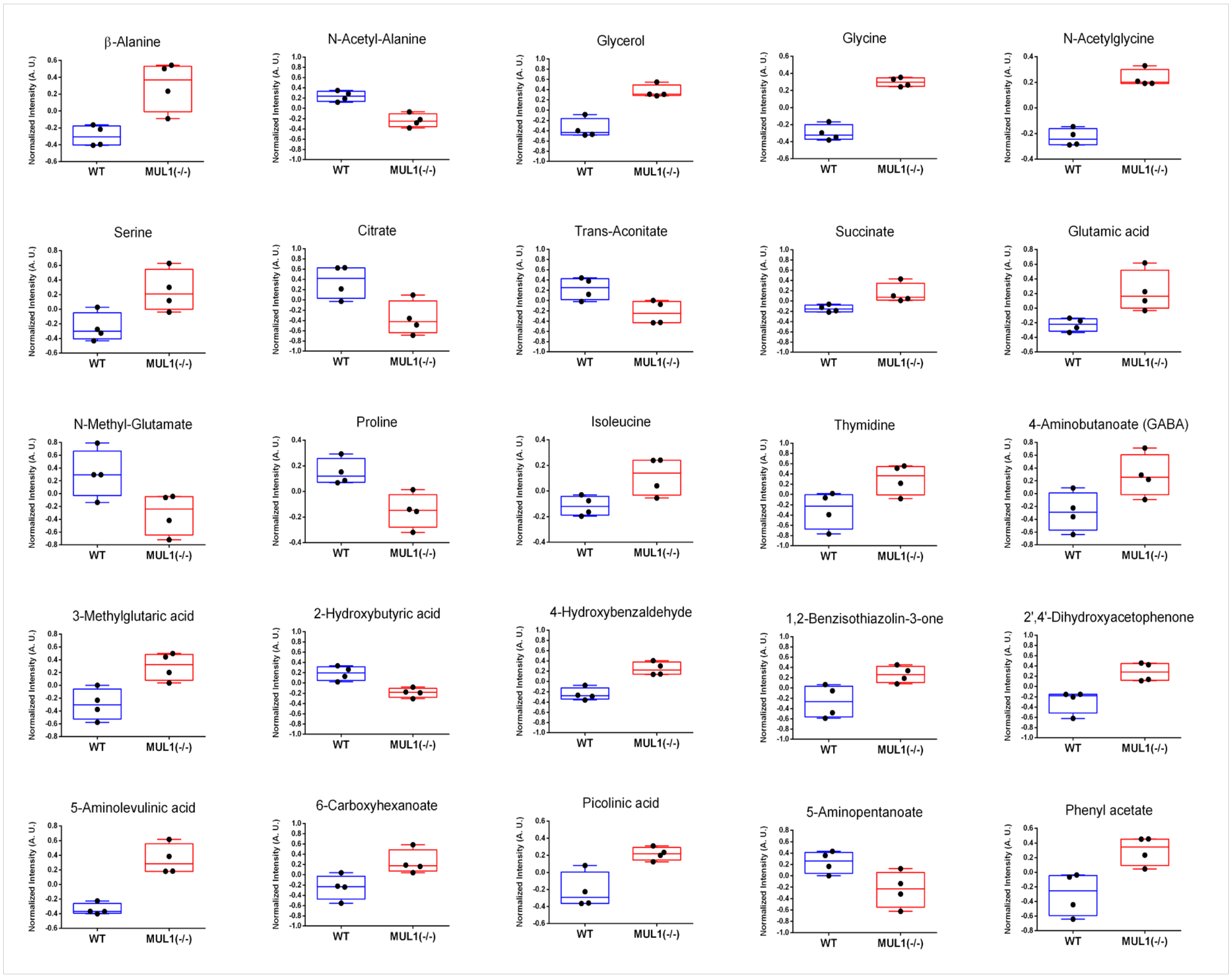

Figure S4. Semi-quantitative metabolomics panel. Box and Whisker plots displayed the differential level of significantly different metabolites between HEK293 WT and MUL1(-/-) cells.

Supplementary Figure S5

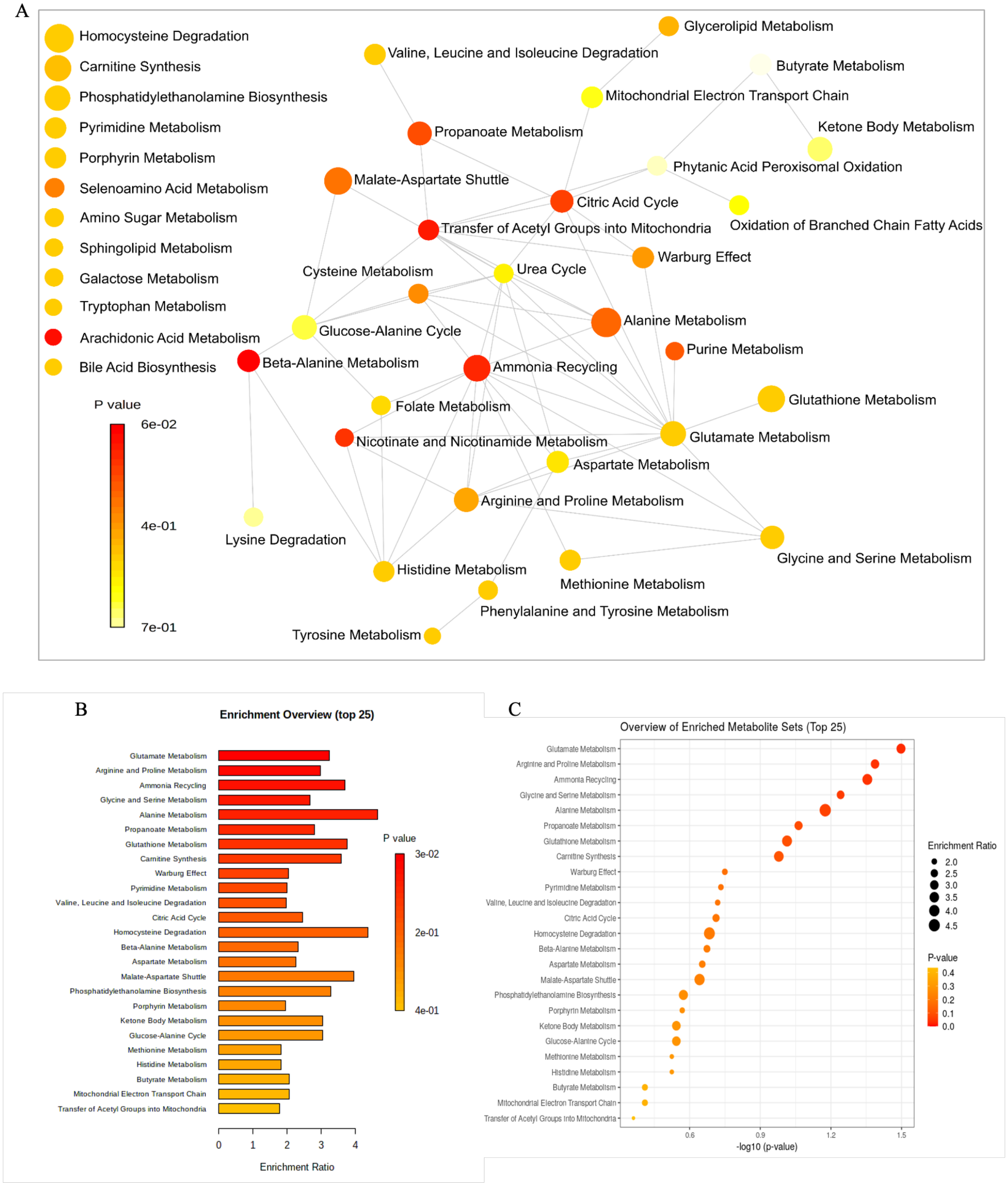

Figure S5. **Metabolite Set Enrichment Analysis. A)** Interactive network of pathways showing the connections between individual pathways. Significantly different Metabolites between HEK293 WT and MUL1(-/-) cells were used for metabolite set enrichment analysis. It is used to investigate if a group of functionally related metabolites are significantly enriched, eliminating the need to preselect compounds based on some arbitrary cut-off threshold. **B)** Top 25 enrichment overview of MSEA. **C)** Dot plot of the enrichment analysis results. The size of the circles per metabolite set represents the Enrichment Ratio and the color represents the *P*-value.
